# Elevated reactive oxygen species can drive the alternative lengthening of telomeres pathway in ATRX-null cancers

**DOI:** 10.1093/nar/gkaf061

**Published:** 2025-02-08

**Authors:** Tomas Goncalves, Siobhan Cunniffe, Tiffany S Ma, Natalie Mattis, Andrew W Rose, Thomas Kent, David R Mole, Helene E B Geiller, Linda van Bijsterveldt, Timothy C Humphrey, Ester M Hammond, Richard J Gibbons, David Clynes, Anna M Rose

**Affiliations:** MRC Molecular Haematology Unit, Weatherall Institute of Molecular Medicine, University of Oxford, Oxford, OX3 9DS, UK; Department of Oncology, University of Oxford, Oxford, OX3 7DQ, UK; Department of Oncology, University of Oxford, Oxford, OX3 7DQ, UK; Department of Paediatrics, University of Oxford, Oxford, OX3 9DU, UK; Department of Physics, Faculty of Natural Sciences, Imperial College, London, SW7 2BW, UK; MRC Molecular Haematology Unit, Weatherall Institute of Molecular Medicine, University of Oxford, Oxford, OX3 9DS, UK; Nuffield Department of Medicine, University of Oxford, Oxford, OX3 7BN, UK; Department of Oncology, University of Oxford, Oxford, OX3 7DQ, UK; Department of Oncology, University of Oxford, Oxford, OX3 7DQ, UK; Department of Oncology, University of Oxford, Oxford, OX3 7DQ, UK; Department of Oncology, University of Oxford, Oxford, OX3 7DQ, UK; MRC Molecular Haematology Unit, Weatherall Institute of Molecular Medicine, University of Oxford, Oxford, OX3 9DS, UK; Department of Oncology, University of Oxford, Oxford, OX3 7DQ, UK; MRC Molecular Haematology Unit, Weatherall Institute of Molecular Medicine, University of Oxford, Oxford, OX3 9DS, UK; Department of Paediatrics, University of Oxford, Oxford, OX3 9DU, UK

## Abstract

The alternative lengthening of telomeres (ALT) pathway is a telomerase-independent mechanism for immortalization in cancer cells and is commonly activated in low-grade and high-grade glioma, as well as osteosarcoma. The ALT pathway can be activated under various conditions and has often been shown to include mutational loss of *ATRX*. However, this is insufficient in isolation and so other cellular event must also be implicated. It has been shown that excessive accumulation of DNA:RNA hybrid structures (R-loops) and/or formation of DNA–protein crosslinks (DPCs) can be other important driving factors. The underlying cellular events leading to R-loop and DPC formation in ALT cancer cells to date remain unclear. Here, we demonstrate that excessive cellular reactive oxygen species (ROS) is an important causative factor in the evolution of ALT-telomere maintenance in ATRX-deficient glioma. We identified three sources of elevated ROS in ALT-positive gliomas: co-mutation of *SETD2*, downregulation of *DRG2*, and hypoxic tumour microenvironment. We demonstrate that elevated ROS leads to accumulation of R-loops and, crucially, resolution of R-loops by the enzyme RNase H1 prevents ALT pathway activity in cells exposed to elevated ROS. Further, we found a possible causal link between the formation of R-loops and the accumulation of DPCs, in particular, formation of TOP1 complexes covalently linked to DNA (Top1cc). We also demonstrate that elevation of ROS can trigger over-activity of the ALT pathway in osteosarcoma and glioma cell lines, resulting in excessive DNA damage and cell death. This work presents important mechanistic insights into the endogenous origin of excessive R-loops and DPCs in ALT-positive cancers, as well as highlighting potential novel therapeutic approaches in these difficult-to-treat cancer types.

## Graphical Abstract

**Graphical Abstract:**
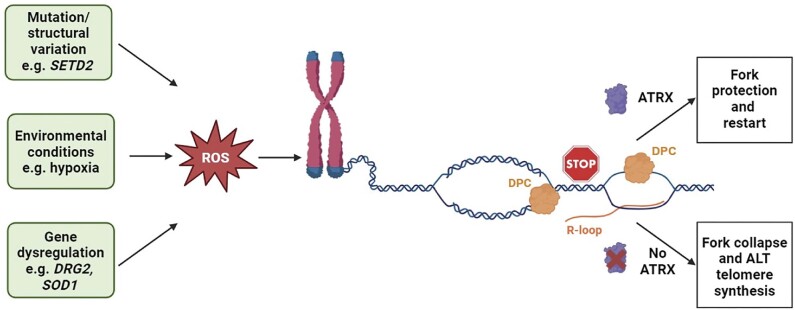

## Introduction

The evolution of cancer involves a complex interplay between genetic, epigenetic, cellular and microenvironmental events, culminating in uncontrolled cell division. These cumulative changes underpin the development of the hallmarks of cancer, including immortalization and genetic instability. Telomeres are nucleoprotein structures found at the end of linear chromosomes. Under normal conditions, telomeres shorten with every round of cell division, until a critical shortening is reached: when DNA damage checkpoints are activated, and the cell undergoes either senescence or programmed cell death. Immortalization is the process by which malignant cells avoid this process, through telomere elongation. In the majority of cancers, this is achieved through re-expression of the enzyme telomerase, but in approximately 10%–15% of adult cancer types (and a higher proportion of paediatric solid tumours), a telomerase-independent pathway is used, termed alternative lengthening of telomeres (ALT) [1]. The ALT pathway is particularly prevalent in cancers which affect children and young adults, such as high-grade glioma (HGG) and low-grade glioma (LGG), soft tissue sarcoma, osteosarcoma, and neuroblastoma [1]. ALT cancers tend to be associated with poor prognosis and, as such, development of novel therapies to specifically target this group of tumours is an area of unmet clinical need [2].

ALT telomere synthesis is a form of aberrant break-induced replication (BIR), where a one-ended DNA double-strand break (DSB) leads to homologous recombination between telomeres [3–6]. There are several molecular features that comprise the ALT phenotype and allow *in vitro* assessment of ALT status. ALT cells have long, heterogeneous telomere length, G2-phase incorporation of EdU at telomeres, abundant circular extrachromosomal telomeric DNA (C-circles and T-circles), and nuclear structures termed ALT-associated promyelocytic leukaemia (PML) bodies (APBs), where telomeres co-localize with PML bodies [7]. One of the central genetic events underpinning ALT cancers is well-established: approximately 90% of ALT-positive cancers lose *ATRX* gene expression, usually due to nonsense truncating mutations [8–10]. In a minority of cases, tumours exhibit loss of DAXX (a binding partner of ATRX) and, more rarely still, ALT-like processes operate in tumours with neither ATRX nor DAXX loss [1, 11]. ATRX is a chromatin remodelling factor that facilitates incorporation of the histone variant H3.3 into defined genomic sites, such as telomeric chromatin [12, 13]. ATRX also has several roles in maintenance of genome stability, such as regulation of noncanonical DNA secondary structures [G-quadruplexes (G4), R-loops], prevention of replication fork stalling, potentiation of fork restart, and the prevention of excessive nucleolytic degradation of stalled forks (reviewed in [14]).

Despite the near-universal loss of *ATRX* in ALT cancers, and capacity to suppress the ALT pathway upon re-expression in established ALT cell lines, isolated loss of the gene is insufficient to induce the ALT phenotype in most cellular systems [15]. As such, in addition to *ATRX* loss, induction and maintenance of ALT activity must require a second genetic, epigenetic, or cellular event. Accumulation of R-loops (a noncanonical DNA structure, composed of an RNA:DNA duplex and a displaced strand of DNA) has been proposed to be one such factor, as depletion of R-loops in ALT cancer cells leads to rapid loss of telomere length and suppression of ALT pathway activity [16–21]. Whilst loss of ATRX has been associated with elevated levels of R-loops, ATRX loss alone is insufficient for ALT-pathway initiation [22, 23]. Formation of R-loops at telomeres has been shown to be a RAD51AP1-dependent process, which increases G4 structure formation and stabilization [24]. Both G4s and R-loops can present a barrier to DNA replication and can cause replication fork stalling [25–27]. Additionally, our recent work demonstrated that formation of both covalent and noncovalent DNA–protein crosslinks (DPCs) are a possible fundamental factor in the induction of ALT, with natural ALT cancers harbouring very high levels of such complexes [28]. DNA-associated proteins – such as the topoisomerases TOP1 and TOP2A – can form covalent DPCs, and other proteins, such as PARP1, can form tightly chromatin-bound noncovalent complexes. These complexes represent a significant source of replicative stress, because, when the progressing replication fork encounters a trapped protein, the fork will stall [29, 30]. In the case of both abnormal DNA structure and DPCs, if the lesion at the stalled fork remains unrepaired, the fork will collapse [29, 30]. In the absence of ATRX, collapse and nucleolytic degradation of the stalled replication fork can occur [31]; this has been proposed to provide the genetic substrate for BIR at telomeres, leading to aberrant recombination and telomere elongation (reviewed in [14]).

Despite strong evidence for the causal role of accumulated R-loops and DPCs in ALT-pathway initiation and maintenance, the endogenous origin of these elevated levels has remained obscure. This study aimed to investigate the origin of increased R-loops and DPCs in ALT cancers, with a particular focus on LGG and HGG.

First, we identified mutational loss of *SETD2* to be prevalent in a subset of ATRX-deficient HGG, and that this change was implicated in ALT-pathway initiation. Similarly, we examined patterns of gene dysregulation in glioma tumour samples and found that downregulation of *DRG2* was strongly associated with *ATRX*-loss in LGG. Crucially, we identified that both genetic changes – SETD2 mutational loss and DRG2 downregulation – led to elevated reactive oxygen species (ROS) levels, and that ALT-pathway activity was decreased by cellular antioxidant therapy. Furthermore, we saw that exposure of cells to hypoxia led to both elevation of cellular ROS and induction of ALT-pathway activity. This data implicates ROS of various sources as causative of ALT-pathway activation in cells lacking ATRX.

Next, we investigated the mechanism by which ROS initiated ALT pathway activity, and whether it was connected to the previously described factors in ALT pathway initiation. We found that various sources of ROS led to accumulation of R-loops, as well as formation of DPCs. We demonstrated that elevated ROS levels acted as a potent trigger for ALT pathway activity in ATRX-null cells, and this process was dependent on both formation of R-loops and DPCs, providing important mechanistic insight into the initiation and maintenance of ALT pathway activity in HGG, LGG and other ALT-positive cancers.

Finally, we considered whether these advances in understanding of ALT pathway mechanism might be of use as a therapeutic strategy in ALT-positive cancer cells. In recent years, research into development of new treatments in ALT cancer has focussed on strategies which increase ALT pathway activity, triggering excessive genetic instability and rapid cell death – the *hyper-ALT* phenotype [19, 20]. Here, we observed that treatment of ALT-positive HGG and osteosarcoma cell lines with ROS-producing agents was a potent strategy for inducing hyper-ALT and could, therefore, represent an exciting new therapeutic approach in these difficult-to-treat cancers.

## Materials and methods

### Cell lines and cell culture conditions

The majority of cell lines used in this study were acquired from ATCC, apart from HeLa LT, which was a gift from Roderick O’Sullivan (University of Pittsburgh, USA), as described in [32]. Cells were maintained in a 5% CO_2_ 37°C incubator with Dulbecco’s modified Eagle’s medium (DMEM) media supplemented with 10% foetal bovine serum, 1% L-glutamine and 1% PenStrep (all Gibco). Cells were split every few days to maintain at a confluency of between 70% and 90% using 0.05% trypsin/ethylenediaminetetraacetic acid (EDTA) (Gibco). Hypoxia treatments were carried out in a Bactron II, Bactronez-2 anaerobic chamber (Shell labs) or a Don Whitley Scientific H35 chamber. For experiments at <0.1% O_2_, cells were plated on glass dishes and were harvested inside the chamber with equilibrated solutions.

### CRISPR-Cas9 knockouts

CRISPR-Cas9 knockout of ATRX was previously performed, as described in [28]. SETD2 CRISPR-Cas9 knockout was performed, as described in [33]. Transfected cells were selected using 0.4 μg/ml puromycin and then sorted into single wells to obtain clones. Combinatorial knockouts were made through sequential knockout of genes. Knockout efficiency was determined by immunoblotting.

### Treatment of cells with chemicals

Cells were treated with these chemicals at the following doses prior to downstream analysis with the assays used in this study: 50–500 μM hydrogen peroxide (H_2_O_2_) (Sigma); 0.5–1.5 mM N-acetylcysteine (Sigma) and 5–50 μM tert-butyl hydroperoxide (t-BHP) (Sigma). Negative controls with the drug diluent only (water or dimethyl sulfoxide (DMSO)) were also carried out with each assay.

### Immunoblotting

Whole cell lysates were prepared with approximately 1 × 10^6^ cells in 100 μl ice-cold lysis buffer [50 mM Tris-HCl (pH 7.4), 100 mM NaCl, 1 mM MgCl_2_, 10% glycerol, 5 mM NaF, 0.2% IgePal-CA630) containing Pierce protease inhibitor tablet (Thermo Fisher Scientific). Lysates were quantified by a NanoDrop and equal concentrations were loaded per lane into precast 4%–12% bis-tris gels with MOPS (3-(N-morpholino)propanesulfonic acid) running buffer (both Thermo Fisher Scientific). Membranes were transferred at a constant current of 30 mA overnight onto polyvinylidene fluoride (PVDF) membranes (Millipore) with NuPage transfer buffer (Thermo Fisher Scientific) supplemented with 10% methanol. The following day, membranes were blocked in 5% milk in PBST [0.1% tween in 1× phosphate-buffered saline (PBS)] and then incubated for 1 h at room temperature with the following primary antibodies in 2.5% milk in PBST: rabbit anti-SETD2 (GeneTex, GTX127905, 1:500), rabbit anti-KAP1 (Abcam, ab10483, 1:5000), rabbit anti-histone H3K36me3 (Abcam, ab9050, 1:1000), mouse anti-superoxide dismutase 1 (SOD1) (Santa Cruz, sc-17767, 1:200), mouse anti-RNase H1 (Santa Cruz, sc-101114, 1:200), rabbit anti-V5 (Cell Signaling, 13202S, 1:1000), rabbit anti-DRG2 (Proteintech, 14743–1-AP, 1:5000), mouse anti-H3 (BioLegend, 819 414, 1:100 000), rabbit anti-ATRX (Abcam, ab97508, 1:1000), mouse anti-ATRX (39F, 1:17), and mouse anti-alpha Tubulin (Abcam, ab7291, 1:50 000). Membranes were then washed three times at room temperature for 10 min with PBST before incubation with the following horseradish peroxidase (HRP)-conjugated secondary antibodies diluted in 2.5% milk in PBST for 1 h at room temperature: rabbit anti-mouse IgG HRP (Sigma, A9044, 1:5000) or goat anti-rabbit IgG HRP (Thermo Fisher Scientific, 31 460, 1:5000). Membranes were again washed three times for 10 min with PBST and membranes were finally developed using SignalFire ECL reagent (Cell Signaling) onto X-ray films (Amersham).

### shRNA (short hairpin RNA) knockdowns

shRNA knockdown of SOD1 was performed using MISSION shRNA TOP1 (Sigma, NM_003286–3990) plasmid DNA, which was lentivirally packaged in house. shRNA knockdown of DRG2 or control shRNA particles was performed using the following lentiviral particles from Santa Cruz: DRG2 (sc-93839-V) and control (sc-108080). Puromycin kill curves were performed to establish the minimum lethal dose for nontransduced cells 48 h post transduction. Cells were seeded into 12-well plates and, the following day, 500 μl of the lentivirus mix (containing 20 μl of lentivirally packaged shRNA, 479 μl of DMEM and 1 μl of polybrene) was added. Seventy-two hours after transduction, cells were held under selection with puromycin (3 μg/ml) for up to 8 days (with media being replaced every 2 days) before being harvested or fixed for downstream assays. For shDRG2 knockdown cells were seeded to clonal density and individual clones were grown up to select clones with good knockdown efficiency. Western blots were performed to validate knockdowns. A negative control, using a scrambled shRNA sequence, was included with each assay.

### siRNA (small interfering RNA) knockdowns

siRNA knockdown of SOD1 was performed using a commercially available ON-TARGETplus siRNA SmartPool (L-008364–00-0005, Dharmacon) along with a nontargeting control pool (D-001810–01-05). Cells were transfected using Lipofectamine RNAiMAX (Thermo Fisher Scientific) to a final concentration of 5 pmol according to manufacturer’s instructions. Seventy-two hours after transfection, cells were harvested for downstream analysis.

### Immunofluorescence/ImmunoFISH

Approximately 50 000 cells were seeded onto 13 mm #00 thickness glass coverslips in 24-well plates. Unless otherwise stated, once ready for staining, cells were pre-permeabilized in 0.5% triton X-100 (Sigma) in PBS for 1 min on ice (increased to 5 min for TOP1cc staining) and then fixed with 4% paraformaldehyde (Thermo Fisher Scientific; 16% diluted to 4% in PBS) for 20 min at room temperature. Fixed cells were briefly washed three times in PBS at room temperature and then permeabilized with 0.5% triton X-100 on ice for 6 min (increased to 20 min for TOP1cc staining). Cells were washed again three times in PBS and then blocked for >1 h in 1% bovine serum albumin (BSA) in PBS. After blocking, fixed cells were incubated for >1 h with the following primary antibodies diluted in 1% BSA in PBS: mouse anti-PML (Santa Cruz, sc-966, 1:300), rabbit anti-TRF2 (Novus Biologicals, NB110-57130, 1:500), mouse anti-TRF2 (Novus Biologicals, NB100-56506, 1:500), mouse anti-Top1cc (from ICE assay kit, TopoGEN, TG1020), mouse anti-53BP1 (Santa Cruz, sc-515841, 1:300), rabbit anti-pRPA-S33 (Bethyl Laboratories, A300-246A, 1:300), mouse anti-γH2AX (Sigma, 05–636, 1:300), mouse anti-RPA32 (Abcam, ab2175, 1:500), and mouse anti-BLM (Santa Cruz, sc-365753, 1:300). Cells were then washed a further four times with PBST and then incubated for >1 h with the following fluorescently labelled secondary antibodies: goat anti-rabbit Alexa Fluor 568 (Life Technologies, A11036, 1:3000), goat anti-mouse Alexa Fluor 488 (Life Technologies, A11029, 1:3000). After another three 5-min washes in PBST, coverslips were mounted onto microscope slides with VectaShield containing DAPI (4',6-diamidino-2-phenylindole) and imaged using a DeltaVision widefield microscope at 60× magnification. Images were z-projected using Fiji ImageJ software and image analysis was performed using CellProfiler [34], with no fewer than 100 cells imaged per repeat.

ImmunoFISH experiments were carried out as described above up until PBST washes after secondary antibody incubation. From this step, cells were post-fixed with 4% paraformaldehyde for 10 min at room temperature. When required (all experiments apart from RPA-ssTel analysis), telomeres were denatured using 3.5 N HCl, the reaction was quenched in ice-cold PBS and then samples washed twice in PBST and 2× SSC (saline sodium citrate). A Cy3-[CCCTAA]_5_ labelled probe was hybridized onto the cells overnight in a 37°C humidified chamber with hybridization buffer (25% formamide, 2 × SSC, 200 ng/μl salmon sperm, 5 × Denhardt’s solution, 50 mM phosphate buffer, 1 mM EDTA). The following day, coverslips were washed three times with 2× SSC at 37°C, followed by two washes in PBST and one wash in PBS. Coverslips were then mounted and imaged as above.

For 8-oxoG staining, cells were fixed with ice-cold methanol:acetone (1:1 ratio) rather than 4% paraformaldehyde. After this, cells were air dried and then treated with 0.05 N HCl for 5 min on ice. Following three washes in PBS at room temperature, cells were treated with 100 μg/ml RNase A in a 150 mM sodium chloride; 15 mM sodium citrate solution. Cells were washed in sequential 3-min washes of PBS, 35% ethanol, 50% ethanol, and 75% ethanol, respectively. DNA was denatured with 0.15 N NaOH in 70% ethanol for 4 min at room temperature and cells were once again washed twice in PBS. After four further 2-min washes consisting of 70% ethanol/4% formaldehyde, 50% ethanol, 35% ethanol, and 1× PBS, samples were treated with 5 μg/ml proteinase K in 20 mM Tris-HCL; 1 mM EDTA; 1× tris-EDTA (pH 7.5) at 37°C for 10 min and then washed another three times in 1× PBS. Cells were then blocked for 1 h in 1% BSA/10% FBS in 1× PBS and then incubated overnight at 4°C with the mouse anti-8-oxoG antibody (Santa Cruz, sc-130914, 1:250). Washes, secondary antibody incubation, mounting and imaging were then all carried out as above.

### ATSA assay

The ATSA (ALT telomere DNA synthesis in APBs), which allows direct visualization of DNA replication at telomeres during G2, representing direct evidence of ALT telomere synthesis, was carried out as described previously, with minor alterations [35]. Approximately 100 000 cells were plated onto coverslips and, the following day, were synchronized in G2 by treatment with 10 μM of the CDK1 inhibitor RO-3306 for 18 h. The synchronized cells were then pulsed with 20 mM EdU for 1 h. Coverslips were then harvested for FISH, as described above, and this was followed by EdU detection using the Click-IT Plus EdU Cell Proliferation Kit with Alexa Fluor 488, according to manufacturer’s instructions (Invitrogen). Cells were then washed for 3 times with 1 × PBST, mounted with DAPI and images were captured with a Deltavision widefield microscope, as described above.

### C-circle assay

Genomic DNA was extracted from approximately 1 × 10^6^ cells using the PureLink genomic DNA extraction kit (Thermo Fisher Scientific) and quantified using a NanoDrop. Between 30 and 120 ng of DNA was amplified by polymerase chain reaction (PCR) (for 8 h at 30°C followed by 20 min at 65°C) in a reaction containing 7.5 U of phi-29 polymerase (New England Biolabs), 0.1% Tween-20, 200 μg/ml BSA (New England Biolabs) and 1 mM each of dTTP, dGTP and dATP (all New England Biolabs) diluted in DNase-free water. Negative controls (without phi-29 polymerase) and positive controls (U2OS genomic DNA (gDNA)) were included in each experiment. Amplified samples were diluted with 2× SSC buffer and transferred onto Zeta-Probe membranes (Bio-Rad) using a slot blot filtration manifold (Bio-Rad BIO-DOT, 48-well). A 2-fold dilution series (4:2:1) was loaded onto each membrane to assess signal linearity of the assays. DNA was then ultraviolet (UV)-crosslinked to the membranes which were then pre-hybridized with DIG Easy Hyb (Roche) for 20 min at room temperature and then incubated in a 37°C hybridization oven for >2 h with a 3′DIG-labelled [CCCTAA]_5_ probe diluted in DIG Easy Hyb to a final concentration of 40 nM. Following incubation with the probe, membranes were briefly washed twice in MS wash buffer (0.1 M maleic acid, 3 M NaCl, 0.3% Tween-20, pH 7.5) and then blocked for 30 min at room temperature in MS blocking buffer (1% milk and 1% BSA in 0.1 M maleic acid, 3 M NaCl, pH 7.5). Anti-DIG-AP Fab fragments (Roche) were added to the MS blocking buffer (1:20 000) and the membranes were incubated for a further 30 min at room temperature. The membranes were then washed three times for >15 min with MS wash buffer and then developed using CDP-Star chemiluminescent substrate solution (Roche) for X-ray film detection. Films were analysed and quantified using ImageJ software. Normalized C-circle levels were expressed as arbitrary units relative to a U2OS reference sample from the same membrane. Experiments were performed on at least two independent DNA samples for each test condition.

### Telomere restriction fragment (TRF) assay

Between 5 and 20 μg of purified genomic DNA was digested at 37°c overnight with 10 U of HinfI and RsaI restriction endonucleases (New England Biolabs) in a reaction containing 1× CutSmart buffer (New England Biolabs) and nuclease-free water. Digested DNA was run on either a 0.8% or 0.5% agarose gel at a 50 V for 18 h and were visualized to ensure equal loading and complete digestion. The gel was incubated with depurinating buffer (0.25 M HCl) for 20 min, denaturing buffer (1.5 M NaCl; 0.5 M NaOH) for 30 min and neutralising buffer (1 M NH4Ac; 1.5 M NaOH) for two 20-min washes, all at room temperature. The gel was transferred overnight to Zeta-Probe nylon membranes (BioRad) through capillary action. The following day, the DNA was UV-crosslinked to the membrane and the membrane was stained using the DIG-probe, as described above. Indicated blots were analysed and quantified using the WALTER online toolset [36].

### Monochrome multiplex quantitative polymerase chain reaction for telomere length analysis

Monochrome multiplex quantitative polymerase chain reaction (qPCR) was carried out as described in [37], with minor modifications. Primer sets used are listed in Supplementary Table S2. Five concentrations of reference genomic DNA purified from HeLa LT were prepared by 3-fold serial dilution (from 150 to 1.85 ng) to generate standard curves for relative quantitation of T/S ratios [the ratio of the telomeric signal compared to a single copy gene (SCG)]. For each sample, 20 ng of genomic DNA was mixed with 0.75 × PowerUp SYBR Green Master Mix (Thermo Fisher Scientific), the relevant forward and reverse primers (300 nM), and water to a final volume of 20 μl per well and analysed using a Thermo Fisher QuantStudio 3 qPCR machine with the following cycle conditions: denaturation for 15 min at 95°C, followed by two cycles of 15 s at 94°C/15 s at 49°C and 32 cycles of 15 s at 94°C/10 s at 62°C/15 s at 74°C with signal acquisition and 10 s at 84°C/15 s at 88°C with signal acquisition. Samples were run in triplicate, and analysis was repeated with three biological replicates. Ct values were extracted using the online Thermo Fisher cloud dashboard and were used to calculate T/S ratios by normalizing the Ct value of the samples to the Ct values of the standard curve. A T/S ratio of >1.0 corresponds to a relative telomere length greater than the HeLa LT controls, while a T/S ratio of <1.0 corresponds to a relative telomere length lower than the controls.

### DCFDA assay

A total of 20 000 cells were plated in a 96-well black cell culture grade plate (Greiner, UK); the following day DCFDA assay was performed according to manufacturer’s instructions using H2DCFDA reagent (Thermo Fisher Scientific). A positive control of cells treated with 400 μM of H_2_O_2_ for 1 h was used as a positive control on each plate; a negative control of 1× PBS was also included on each plate. Fluorescence (485/530 nm) was read on ClarioStar plate reader; readings were normalized for cell number.

### CellRox green quantification of cellular ROS

During the last 10 min of treatment, cells grown on coverslips were incubated in the dark at 37°C with media containing 5 μM of CellRox Green (Invitrogen) then fixed with 4% paraformaldehyde. Coverslips were mounted with DAPI mounting media and visualized on a LSM780 confocal microscope (Carl Zeiss Microscopy Ltd), using a 63×/1.40 Oil DIC M27 Plan-ApoChromat objective lens. DAPI was excited with laser line 405 nm and emission collected between 410 and 495 nm. The fluorescent CellRox Green was excited with laser line 488 nm and emission collected between 495 and 534 nm. Nuclear intensity of fluorescence was quantified using ImageJ software.

### CellTiter glo assay

Cells were seeded onto opaque 96-well plates at 100–500 cells in 100 μl of the culture media per well. After 6–24 h, an equal volume of the drug-containing media was added at the desired final concentrations and the cells were left for 5 days. Cell proliferation was assessed using CellTiter-Glo 2.0 Reagent (ProMega). Briefly, the plate and the reagent were equilibrated at the room temperature for 5 min, the media was removed from the plate, and 100 μl of the diluted reagent (1:6 in PBS) was added to each well. After 10 min, the plate was read via the ProMega GloMax Luminometer to assess the quantity of the metabolically active cells. The IC_50_ values were derived by fitting the dose-response data into the curves via GraphPad Prism.

### Clonogenic survival assay for H_2_O_2_ treatment

Approximately 500 cells were seeded in triplicate into 6-well plates for each condition. The following day, cells were treated with varying doses of H_2_O_2_ (50–500 μM). After 24 h, the H_2_O_2_ was replaced and after 48 h the drug was removed and replaced with fresh media. The cells were then allowed to grow for a further 10 days, to allow colonies to form. Colonies were then fixed and stained using a 50% methanol; 10% glacial acetic acid; 0.1% Coomassie Brilliant Blue R-250 (*w/v*) solution. Following staining, plates were washed in water and colonies were then counted by hand, excluding colonies <1 mm in width.

### Clonogenic survival assay for hypoxia

Cells were trypsinized into a single-cell suspension and counted. A total of 250–500 cells (depending on the plating efficiency of the cell line) were plated into each well in 6-well plates, with three technical replicates per treatment. Cells were left to settle for 2–3 h before hypoxia treatment (<0.1% O_2_ for 6 h). After treatment, the cells were left to form colonies for 7–9 days in a humidified incubator at 37°C. The media was carefully removed, and crystal violet (0.5% *w/v*) in 50% methanol and 20% ethanol was added to the wells to stain and visualize the colonies. Colonies containing at least 50 cells were counted using a colony counter.

### RNase H1 overexpression

Lentivirus-based constructs for overexpression of wildtype RNase H1 and the catalytically dead D210N mutant in HeLa LT cells were generated using InFusion cloning to insert the NLS-RNaseH-V5 sequences from plasmids ppCAG_RNaseH1_WT (Addgene #111 906) and ppCAG_RNaseH1_D210N (Addgene #111 904) into the pLeGO-C2 backbone (Addgene #27 339). The ATRX/SETD2 double knockout cells were transduced with the lentivirus and left for 14 days before ALT assays were carried out as described above. Efficient overexpression was assessed by immunoblot.

### Telomeric R-loop detection by S9.6 DNA–RNA immunoprecipitation

R-loop DNA–RNA immunoprecipitation (DRIP) experiments were performed using the S9.6 antibody as described in [22], with minor modifications. At least 3 × 10^7^ cells were harvested, washed in 1× PBS and resuspended in cell lysis buffer (0.5% NP-40, 85 mM KCl, 5 mM PIPES, 1× cOmplete protease inhibitor cocktail). Samples were incubated on ice for 10 min and then centrifuged to pellet the nuclei. Pelleted nuclei were then resuspended in nuclear lysis buffer [1% sodium dodecyl sulphate (SDS), 25 mM Tris-HCl (pH 8.0), 5 mM EDTA, 1× cOmplete protease inhibitor cocktail], incubated on ice for 10 min and were then treated with 0.5 mg/ml Proteinase K overnight at 45°C. The following day, gDNA isolation buffer (3 M potassium acetate, 11.5% glacial acetic acid) was added to the samples which were then spun down to remove debris and the supernatant was collected; 1× volume of isopropanol was added to the samples which were then pelleted and rinsed in 70% ethanol before being air dried and resuspended in nuclease free water. The DNA was quantified and then split into two samples (with and without RNase H1 treatment), each containing >35 μg of DNA. For the negative DRIP control, the DNA was treated with 500 U of RNase H1 (NEB) overnight at 37°C, while for the DRIP sample the DNA was incubated with water overnight at 37°C.

The following day, the samples were diluted in IP dilution buffer [0.001% SDS, 1.1% Triton-X 100, 120 μM EDTA, 16 mM Tris-HCl (pH 8.0), 160 mM NaCl, 1× complete protease inhibitor cocktail] and sonicated at 4°C at high power for 10 min with 30 s on and 30 s off cycles. Samples were spun down and the supernatant was taken and diluted with further IP dilution buffer, with 3% taken as the input sample. The remaining sample was pre-cleared with Protein A Dynabeads for 1 h at 4°C, and the samples were then split in two, with one sample containing the S9.6 antibody (1:150) and the other only beads. The samples were then incubated overnight at 4°C.

The following day, pre-blocked beads were added to the samples and incubated at 4°C for 2 h. The beads were then washed with Buffer A (20 mM Tris-HCl, pH 8.0, 2 mM EDTA, 0.1% SDS, 1% Triton X-100, 165 mM NaCl), Buffer B (20 mM Tris-HCl, pH 8.0, 2 mM EDTA, 0.1% Triton X-100, 550 mM NaCl) and Buffer C (10 mM Tris-HCl, pH 8.0, 1 mM EDTA, 1% NP-40, 0.01% Sodium Deoxycholate, 250 mM LiCl) once each at 4°C, followed by three washes with Buffer D (10 mM Tris-HCl, pH 8.0, 1 mM EDTA) at 4°C and one wash with Buffer D at room temperature. The washed beads were eluted in freshly made IP elution buffer (1% SDS, 100 mM NaHCO_3_) and treated with 20 μg/ml proteinase K for 1 h at 45°C. Following this, the DNA was purified using the Qiagen PCR-purification kit and the eluted DNA was analysed by slot blotting and probing using a DIG-tagged telomere probe, as described above.

### Nuclear R-loop detection by RNase H1^D210N^ mutant

R-loop detection using the RNase H1^D210N^ mutant was carried out as previously described in [38], with minor alterations. Approximately 100 000 cells were seeded out on glass coverslips and, the following day, were transduced with V5-tagged RNase H1^D210N^, as described above. Twenty hours later, cells were permeabilized and fixed, as described above. Immunofluorescence staining was carried out with anti-V5 antibody (Cell Signaling, 13202S) and coverslips were visualized on a widefield DeltaVision microscope. Nuclear intensity of V5-tagged RNase H1^D210N^ was determined using Cell Profiler software.

### Reverse transcription quantitative polymerase chain reaction

RNA was extracted from approximately 1 × 10^6^ cells using the PureLink RNA mini kit (Thermo Fisher Scientific). A total of 1 μg of RNA was treated with DNase I for 30 min at 37°C and the reaction was then stopped by the addition of EDTA to a final concentration of 5 mM and heating to 65°C for 10 min. The DNA-free RNA was then reverse transcribed to complementary DNA (cDNA) using 200 U of SuperScript IV reverse transcriptase (Thermo Fisher Scientific) and 50 μM random hexamer oligos, according to manufacturer instructions. cDNA was diluted to 200 μl with nuclease-free water. Initially, screens were performed using TaqMan™ Array Human Antioxidant Mechanisms array plate (Applied Biosystems, catalogue number: 4 414 119) using TaqMan Gene Expression Mastermix (Applied Biosystems) according to manufacturer’s instructions.

Further confirmation of gene dysregulation was performed using targeted primers and SYBR Green: for each reverse transcription quantitative polymerase chain reaction (RT-qPCR) reaction, 5 μl of cDNA was mixed with 0.75× PowerUp SYBR Green Master Mix (Thermo Fisher Scientific), the primers (300 nM) and water to a final volume of 20 μl per well and analysed using a Thermo Fisher QuantStudio 3 qPCR machine with the following cycle conditions: 95°C for 15 min, followed by 40 cycles of 10 s at 95°C, 20 s at 60°C, and 20 s at 72°C. Melt curve analysis was performed immediately thereafter. Primers used are listed in Supplementary Table S2. For both TaqMan and SYBR Green analysis, expression of genes was normalized to GAPDH expression using the ΔCT method and data is presented as ΔΔCT, comparing ATRX-wildtype controls versus the samples of interest. Averages were calculated from at least three biological replicates, each run in duplicate or triplicate.

### Analysis of TCGA dataset

Genomic and transcriptomic data were downloaded from The Cancer Genome Atlas (TCGA) online repository (https://portal.gdc.cancer.gov/projects/TCGA). For each dataset analysed, samples were sorted according to *ATRX*-mutation status, with nonsense/frameshift mutations considered ATRX-mutant, while all other changes were considered wildtype. The RNA transcript per million data was extracted and compared between the two groups for all protein-coding. Statistical analysis was performed and the calculated *P*-values were converted into false discovery rate (FDR) values using the Benjamini–Hochberg procedure. The volcano plot was generated using GraphPad Prism software. Stringent criteria were used to define significance of differential gene expression: a fold change of log_2_ −0.5 < x < 0.5 was considered nonbiologically relevant and FDR values of >0.005 were considered nonsignificant.

Individual records containing RSEM (RNA-seq by expectation-maximization) normalized read counts (Illumina HiSeq) for mRNA expression were also downloaded from the LGG dataset, as well as other indicated datasets, which were log_2_ transformed to plot graphs, comparing ATRX mutant and ATRX wildtype samples. Estimated CNVs were predicted using GISTIC 2.0 (Genomic Identification of Significant Targets in Cancer). The cBioPortal for Cancer Genomics (http://cbioportal.org) was used to visualize the TCGA datasets.

### Quantification and statistical analysis

Statistical analysis was carried out using either GraphPad Prism 9 (GraphPad Software Inc.) or Social Science Statistics calculators (https://www.socscistatistics.com/). Unpaired *t*-tests were used to compare two groups and one-way ANOVA with Welch correction was used to compare more than two groups (both parametric data). Kruskall–Wallis test was used for nonparametric unpaired data. Chi-squared test and Fisher’s exact test were used for contingency table analysis. Linear regression was used for correlation analysis. Sample sizes and *P*-values are shown in the figure legends and significance was considered as: *P < .05, **P < .01, ***P < .001, and ****P < .0001. ns denotes no significance. 

## Results

### Concurrent loss of SETD2 and ATRX induces markers of the ALT pathway

Previous studies have uncovered mutations in various genes that frequently co-occur with *ATRX* mutations in ALT-positive cancers, such as *TP53*, *IDH1* and *H3F3A*, making them candidates for promoting ALT activity [10, 39–41]. One such study analysed a cohort of paediatric HGGs and found that approximately one-third harboured mutations in ATRX; 17.6% of these *ATRX*-mutant samples had co-mutation in *SETD2* and these mutations were mutually exclusive with *H3F3A* mutations [42].

SETD2 is the best studied H3K36 trimethylase in humans and its loss leads to a significant decrease in global H3K36me3 levels. SETD2-mediated H3K36me3 is believed to play a number of important roles in genome stability (reviewed in [43]). Interestingly, *H3F3A* mutations have been shown to be able to re-direct histone lysine methyltransferases – such as SETD2 – from modifying K36 [44]. However, unlike *H3F3A* and *IDH1* mutations, there is little evidence in the literature on a potential role of SETD2 loss in ALT induction, and therefore we considered that this warranted further investigation.

First, we validated the association between *ATRX* and *SETD2* mutations using the PeCan dataset for paediatric glioma (both high-grade and low-grade). Our analysis showed that 7.7% of *ATRX*-mutant samples also harboured a *SETD2* mutation [45]. It was noted that *SETD2* is located at 3p21.31, which commonly shows copy number variant (CNV) loss in a range of tumours [46–48]. Although CNV loss does not always correlate with decreased gene expression, a study demonstrated that *SETD2* was one of 81 tumour suppressor genes in which decreased gene expression appeared to be induced by CNV loss [48]. We confirmed that in the TCGA LGG and HGG datasets, CNV loss of SETD2 was associated with decreased mRNA expression compared to diploid samples (Supplementary Fig. S1A). Importantly, SETD2 CNV loss was significantly more common in *ATRX*-mutant glioma (14.4%) as compared to *ATRX*-wildtype glioma (7.9%) (Supplementary Fig. S1B and C). Additionally, while the sample size was small, when the analysis was restricted to only HGG samples, it was found that three of the four samples with *SETD2* driver mutations also carried an *ATRX* mutation. (Supplementary Fig. S1D and E). Interestingly, all three samples were also *TP53* mutant and *H3F3A* wildtype (Supplementary Fig. S1D). Furthermore, re-analysis of data from a published CRISPR knockout screen revealed that SETD2 is a selective vulnerability of ALT-positive cancers (Supplementary Fig. S1F and G) [49]. Given these findings, it was considered whether SETD2 loss might play a role in ALT activity.

**Figure 1.**
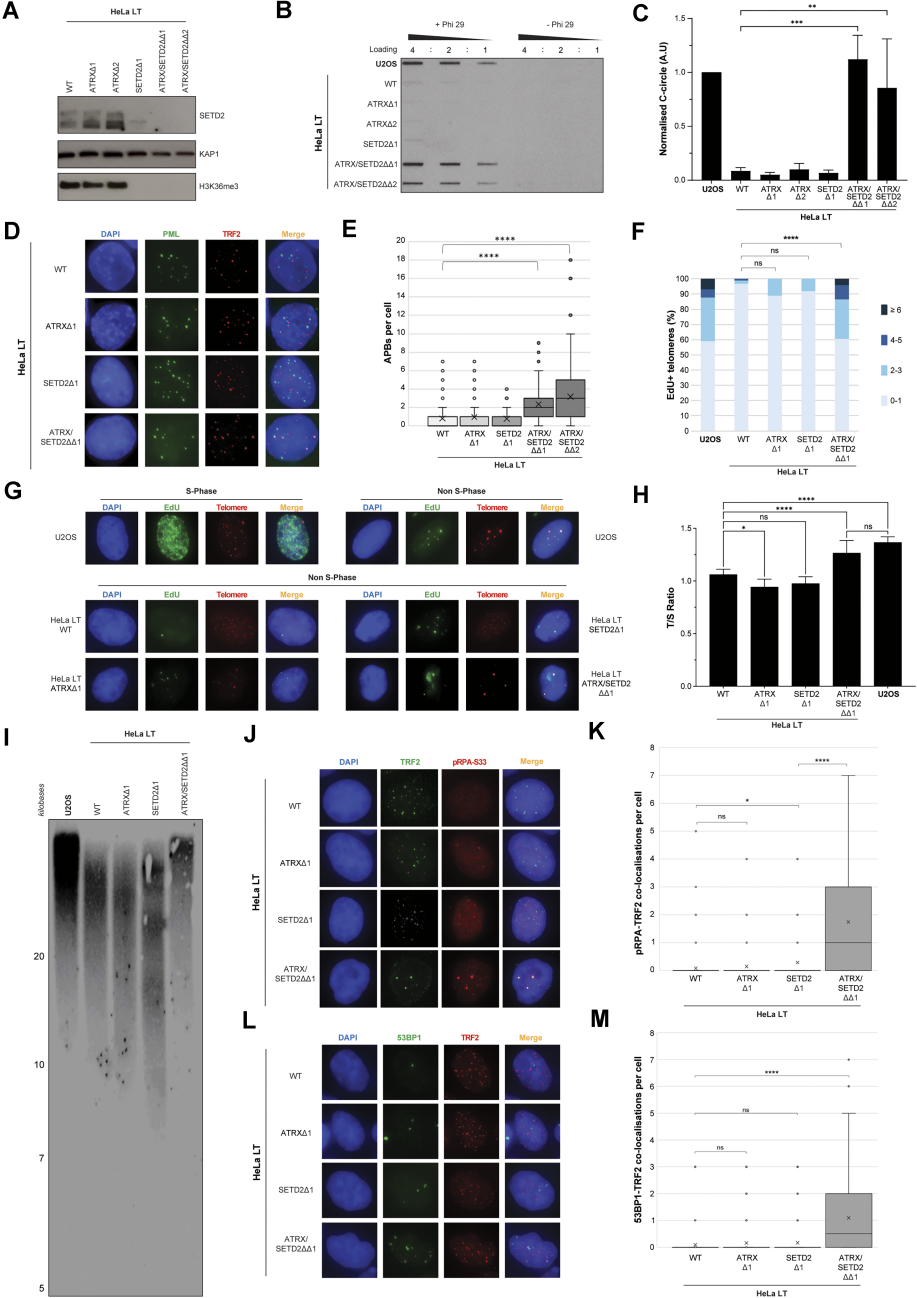
Concurrent loss of *SETD2* and *ATRX* induces markers of the ALT pathway. (**A**) Immunoblot confirming complete loss of H3K36me3 mark upon knockout of SETD2. KAP1 was used as a loading control. (**B**) Representative C-circle assay blot showing that combined loss of ATRX and SETD2 led to strong induction of C-circles. (**C**) Quantification of panel (B), unpaired *t*-test, *n* = 3. (**D**) Representative immunofluorescence images showing that combined loss of ATRX and SETD2 led to formation of APBs. (**E**) Quantification of panel (D), >100 cells analysed for each cell line, Kruskall–Wallis test. (**F**) Cells were synchronized in G2 using CDK1i. At least 100 cells for each condition were divided into four groups (0–1, 2–3, 4–5, and ≥6) depending on the number of EdU + telomeres per cell. (**G**) Representative images for panel (F) showing the localization of EdU and telomeres in G2-synchronized cells. (**H**) qPCR-based analysis, measuring telomere signal intensity over the SCG beta-globin, one-way ANOVA, *n* = 3. (**I**) Representative TRF-based Southern blot assay, showing telomere length distribution in the indicated cell lines. Telomere length heterogeneity was also observed in two further independent clones, as well as in a second replicate for this clone. (**J**) Representative immunofluorescence images showing co-localization between TRF2 and pRPA-S33 upon loss of ATRX and SETD2. (**K**) Quantification of panel (J), >100 cells analysed for each cell line, Kruskall–Wallis test. (**L**) Representative immunofluorescence images showing co-localization between 53BP1 and TRF2 upon loss of ATRX and SETD2. (**M**) Quantification of panel (L), >100 cells analysed for each cell line, Kruskall–Wallis test.

The telomerase positive HeLa LT cell line is amenable to ALT pathway induction under certain conditions of increased replication stress, as demonstrated by us and others [28, 32]. To further understand the role of SETD2 in ALT induction, CRISPR/Cas9-mediated knockouts of SETD2 were generated in either the presence or absence of ATRX in the HeLa LT cell line. Loss of SETD2 activity in the knockout clones was confirmed by global loss of H3K36me3 (Fig. 1A). Loss of either *ATRX* or *SETD2* alone was insufficient to elicit markers of ALT, however, combined ATRX/SETD2 loss induced multiple markers of ALT-pathway activity, including C-circles, APBs and single-stranded telomeric DNA coated by RPA (RPA-ssTel) (Fig. 1B and E and Supplementary Fig. S2A and B).

To establish that the observed markers correlated with ALT-telomere synthesis, we analysed EdU incorporation in non S-phase cells [35]. This analysis demonstrated that there was a significant increase in the number of EdU + telomeres in the ATRX/SETD2 double knockout cells (Fig. 1F and G). Interestingly, when carrying out this analysis, we noted that the double knockout cells displayed large ultrabright telomeric foci, which has previously been demonstrated to be a hallmark of telomere synthesis in ALT-positive cells [50]. Denaturing telomere FISH analysis in asynchronous cells confirmed that the double knockouts displayed a significant increase in median telomere intensity, along with a striking increase in heterogeneity of the signal, another known hallmark of ALT (Supplementary Fig. S2C and D). To confirm this observation, we also analysed telomere length by qPCR and the telomere restriction fragment (TRF) assay – these demonstrating a robust increase in telomere signal and length in the double knockout cells compared to the wildtype or single knockouts (Fig. 1H and I and Supplementary Fig. S2E and F). Two further independent double knockout clones were also tested. These independent clones showed increased telomere length heterogeneity, with both significantly longer and shorter telomeres present – this being consistent with ALT telomere maintenance mechanisms (Supplementary Fig. S2G). The observed telomere length and heterogeneity had a very similar profile to the archetypal ALT-positive U2OS cell line. ALT pathway activity has been shown to be dependent on the BLM helicase [51, 52]. Upon double knockout of ATRX and SETD2, we observed strong recruitment of BLM to telomeres, further confirming true ALT-pathway initiation (Supplementary Fig. S2H and I). Taken together, these data strongly suggest that the induction of ALT markers is associated with an increase in telomere synthesis and *bona fide* activation of the ALT pathway.

Work in the last few years has characterized the ALT pathway as a replication stress-associated mechanism, initiated through replication fork collapse and the generation of a one-ended DSB [53]. We confirmed that induction of ALT in the double knockout cells was associated with elevated levels of telomeric replication stress, as measured by the accumulation of RPA32 phosphorylated at serine 33 at telomeres (pRPA-S33) (Fig. 1J and K). Elevated levels of replication stress can lead to the recruitment of various DNA damage response factors to telomeres, known as telomere dysfunction-induced foci (TIF). It has previously been shown that ALT telomeres display high levels of spontaneous TIFs [54]; indeed, we found that the double knockouts had high levels of TIFs, as measured by 53BP1 and γH2AX recruitment to telomeres (Fig. 1L and M and Supplementary Fig. S2J and K).

### Loss of SETD2 leads to elevated cellular ROS levels, promoting ALT activity

Interestingly, recent studies have associated SETD2 with ROS homeostasis and metabolism. In mice, Setd2 loss has been associated with elevated ROS levels, due to H3K36me3-mediated epigenetic dysregulation of a number of antioxidant genes [55]. Further, SETD2 has also been shown to play a role in the repair of oxidative DNA damage, such as 8-oxoguanine (8-oxoG) [56]. Loss of SETD2 has also recently been shown to increase lipid peroxidation and ROS levels in clear cell renal carcinoma [57].

**Figure 2.**
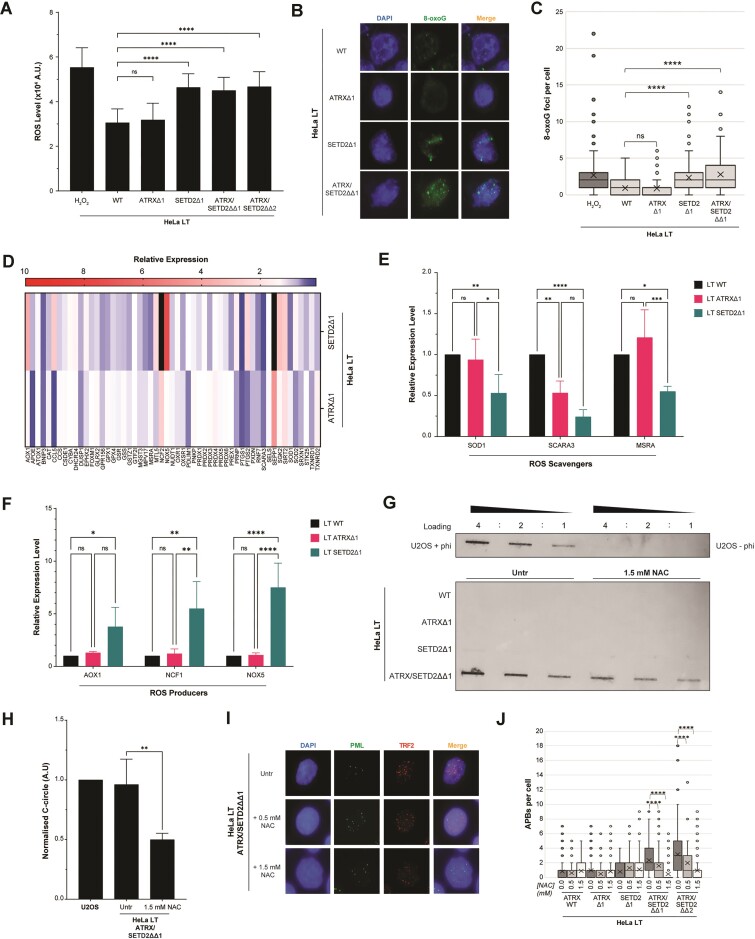
ALT induction through SETD2 loss is due to elevated cellular ROS levels. (**A**) DCFDA assay demonstrated elevated levels of ROS in SETD2 knockout cells, 2 biological replicates and 20 technical replicates, unpaired *t*-test. (**B**) Representative immunofluorescence images showing levels of 8-oxoG lesions increases in SETD2 knockout cells. (**C**) Quantification of panel (B), >100 cells analysed for each cell line, Kruskall–Wallis test. (**D**) Heatmap of a panel of anti-oxidant genes (TaqMan™ Array Anti-Oxidant Mechanisms) shows strong differential expression upon genetic loss of *SETD2*; the effect was much more striking than that observed with the loss of *ATRX*. Data from three biological replicates. (**E**, **F**) Relative gene expression of selected ROS-scavenging (**E**) and ROS-producing (**F**) genes in *SETD2*-knockout cells. (**G**) Representative C-circle blot, showing that the observed induction of C-circles in ATRX/SETD2 knockout cells is reduced upon treatment with 1.5 mM of the anti-oxidant NAC for 48 h. (**H**) Quantification of panel (G), unpaired *t*-test, n = 3. (**I**) Representative immunofluorescence images showing that APB levels in ATRX/SETD2 knockout cells are reduced upon treatment with 0.5 or 1.5 mM of the anti-oxidant NAC for 48 h. (**J**) Quantification of panel (I), >100 cells analysed for each condition, Kruskall–Wallis test.

Corroborating these findings, we found that loss of SETD2 in our cellular system was associated with elevated total ROS levels (Fig. 2A). Levels of 8-oxoG were also analysed; this showed that SETD2 loss increased levels of 8-oxoG, but that concomitant loss of both ATRX and SETD2 did not elevate this further, while ATRX loss in isolation did not induce 8-oxoG lesions (Fig. 2B and C).

Gene expression array assay revealed dysregulation of many key anti-oxidant pathway genes, including downregulation of key ROS scavengers (such as SOD1, SCARA3, and MSRA) and upregulation of ROS producers (such as AOX1, NCF1, and NOX5) (Fig. 2D–F and Supplementary Table S1). It was then asked whether the activation of ALT in the double knockouts was due to this increase in ROS levels upon loss of SETD2. Crucially, when the cells were treated with the antioxidant N-acetyl cysteine (NAC), the previously observed induction of ALT-pathway activity upon combined ATRX and SETD2 loss was ameliorated, indicating that the induction of ALT was, at least in part, a direct consequence of elevated ROS (Fig. 2G–J).

### Direct induction of ROS induces ALT in ATRX-deficient cells

It was next tested whether direct induction of ROS could lead to markers of the ALT phenotype in ATRX-null cells. There are a number of ways in which cellular ROS levels can be increased, with one of the most commonly used methods being exposure to hydrogen peroxide (H_2_O_2_), which releases hydroxyl free radicals, produced through the Fenton’s reaction between H_2_O_2_ and Fe^2+^ ions [58, 59].

**Figure 3.**
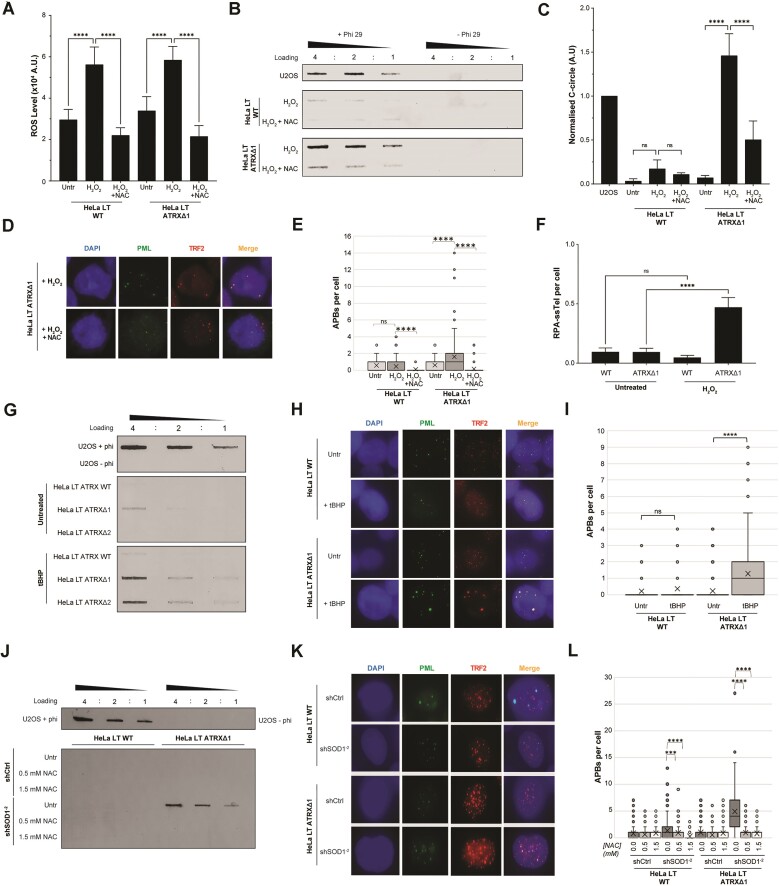
Induction of ALT in ATRX-null cells by treatment with H_2_O_2_, tBHP or through SOD1 depletion. (**A**) DCFDA assay demonstrated elevated ROS levels in cells treated with 100 μM H_2_O_2_ for 48 h, which was reversed by concurrent NAC treatment, 2 biological replicates and 20 technical replicates, unpaired *t*-test. (**B**) H_2_O_2_ treatment induced production of C-circles in ATRX-mutant cells only; this effect was reduced by co-treatment with the anti-oxidant NAC. (**C**) Quantification of (B), unpaired *t*-test, *n* = 3. (**D**) Representative images showing that H_2_O_2_ treatment induced production of APBs in ATRX-null cells only; this effect was ameliorated by co-treatment with the anti-oxidant NAC. (**E**) Quantification of panel (D), >100 cells analysed for each condition, Kruskall–Wallis test. (**F**) H_2_O_2_ treatment induced RPA-ssTEL foci in ATRX-mutant cells, >100 cells analysed for each condition, unpaired *t*-test. (**G**) Representative blot showing induction of C-circles upon 10 μM tBHP treatment for 48 h in the ATRX-null cells only. (**H**) Representative images showing that tBHP treatment induced production of APBs in ATRX-null cells only. (**I**) Quantification of panel (H), >100 cells analysed for each condition, Kruskall–Wallis test. (**J**) Representative blot showing that depletion of SOD1 leads to induction of C-circles specifically in ATRX-null cells. This induction was prevented by concurrent NAC treatment. (**K**) Representative images showing that SOD1 depletion induced production of APBs in ATRX-null cells only. (**L**) Quantification of panel (K), >100 cells analysed for each condition, Kruskall–Wallis test.

Treatment of the cells with 100 μM of H_2_O_2_ for 48h led to a robust increase in ROS levels, and this increase was prevented upon co-treatment with NAC (Fig. 3A). Treatment with H_2_O_2_ showed that ATRX-deficient HeLa LT cells had increased sensitivity to H_2_O_2_ (Supplementary Fig. S3A). This increased sensitivity was associated with robust induction of multiple ALT hallmarks (Fig. 3B–F and Supplementary Fig. S3B). Importantly, these were reversed upon concurrent treatment with NAC (Fig. 3B–E).

To confirm these results, a second agent that can increase cellular ROS levels – the organic peroxide t-BHP – was used (Supplementary Fig. S3C). Treatment of the HeLa LT ATRX-null cells, but not the ATRX wildtype cells, with 10 μM of t-BHP for 48-h led to an induction of C-circles and a significant increase in APB levels versus untreated cells (Fig. 3G–I). This reinforces the results seen with H_2_O_2_ treatment, suggesting that high ROS levels can induce an ALT-like phenotype in the absence of ATRX.

SOD1 is a well-characterized, potent anti-oxidant protein which catalyses the conversion of superoxide radicals to H_2_O_2_ and dioxygen. We therefore sought to assess the effect of SOD1 loss in our cellular system. *SOD1* was knocked down using two separate shRNA sequences in HeLa LT cells (Supplementary Fig. S3D). The level of ROS was assayed, confirming that knockdown of *SOD1* caused an accumulation of ROS (Supplementary Fig. S3E). *SOD1* knockdown resulted in significantly reduced cellular viability in *ATRX*-null clones, as compared to wildtype cells (Supplementary Fig. S3F). The observed reduced viability was accompanied by induction of ALT markers, specifically in *ATRX*-null cells, and this effect was ameliorated by NAC co-treatment (Fig. 3J–L).

### Downregulation of *DRG2* is associated with induction of ALT and generation of ROS

**Figure 4.**
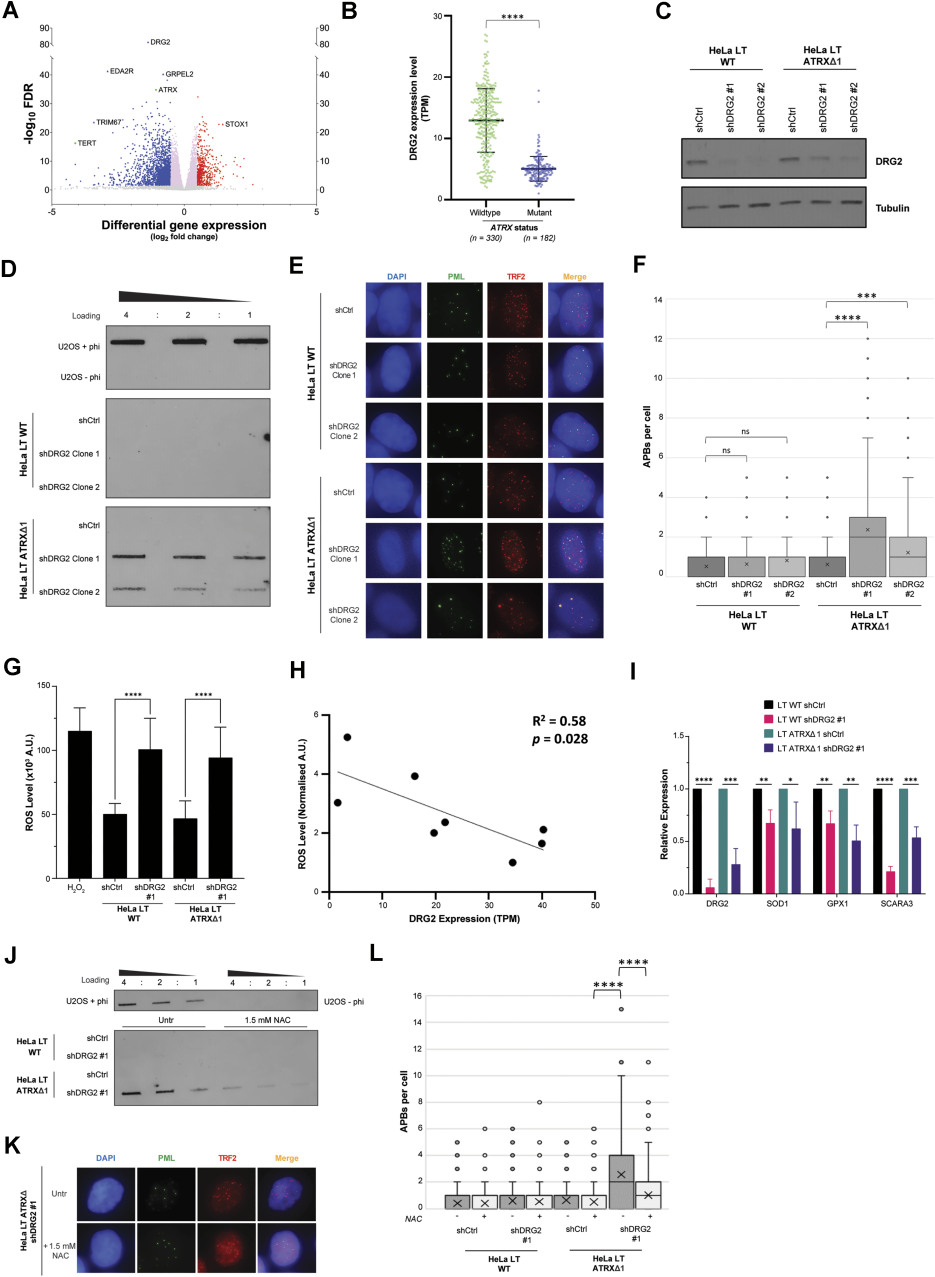
Downregulation of DRG2 is associated with generation of ROS and induction of ALT. (**A**) Volcano plot of DEGs between *ATRX*-wildtype and *ATRX*-mutant LGG, using RNA-seq data. Data presented as log_2_ fold change gene expression and −log_10_ FDR. Stringent criteria were used to define significance: fold change of log_2_ −0.5 < x < 0.5 was considered nonsignificant (pink central dots) and *P*-values of >.005 were also considered nonsignificant (grey). Significantly downregulated genes are highlighted in blue and significantly upregulated genes in red. (**B**) *ATRX*-mutant LGG were found to have significantly lower expression of DRG2 as compared to *ATRX*-wildtype tumours. (**C**) Immunoblot confirming downregulation of DRG2 upon shRNA treatment. Tubulin was used as a loading control. (**D**) Representative blot of C-circle analysis in ATRX-wildtype and ATRX-knockout HeLa LT cells upon silencing of DRG2, showing C-circle induction only in the ATRX-null cells. Similar results were seen in three biological repeats. (**E**) Representative images showing that DRG2 depletion induced production of APBs in ATRX-null cells only. (**F**) Quantification of panel (E), >100 cells analysed for each condition, Kruskall–Wallis test. (**G**) Knockdown of DRG2 was associated with elevated levels of ROS, as measured by the DCFDA assay, 2 biological replicates and 20 technical replicates. (**H**) Scatterplot showing that DRG2 expression level (measured by RNA-seq from Protein Atlas), correlates with level of total intracellular ROS (measured by DCFDA) in a variety of ALT-positive cell lines. (**I**) Knockdown of DRG2 was associated with reduced expression of the indicated antioxidant genes as measured by RT-qPCR across three biological replicates. Primers used are listed in Supplementary Table S2. (**J**) Representative blot showing that the observed C-circle induction in the ATRX-null cells depleted of DRG2 is reversed by 1.5 mM NAC treatment for 48 h. (**K**) Representative images showing that APB levels are reduced in the DRG2-depleted ATRX-null cells with concurrent 1.5 mM NAC treatment for 48 h. (**L**) Quantification of panel (K), >100 cells analysed for each condition, Kruskall–Wallis test.

Given that elevated ROS appeared to be a robust inducer of ALT activity in ATRX-null cells, it was next considered whether any other genetic events which naturally occur concurrently with *ATRX* mutations in tumours that are frequently ALT-positive might exert their effect via generation of ROS. The RNA sequencing data from the LGG dataset within (TCGA-LGG) was analysed, with comparison of protein-coding gene expression between *ATRX*-mutant samples (*n* = 183) and *ATRX*-wildtype samples (*n* = 331) (Fig. 4A). Interestingly, many of the differentially expressed genes (DEGs) have previously been described to have functions in ROS homeostasis, including *GRPEL2*, *EDA2R*, *TRIM67*, and *STOX1* [60–65].

One of the DEGs, developmentally regulated GTP-binding protein 2 (*DRG2*), was a particularly striking outlier, and was consistently downregulated in *ATRX*-mutant low grade glioma (Fig. 4B). Further, ATRX loss was significantly associated with *DRG2* downregulation in several other tumour types which are frequently ALT-positive, such as HGG, osteosarcoma, paraganglioma, and phaeochromocytoma (Supplementary Fig. S4A). Importantly, there was no association between *DRG2* expression level and *ATRX* loss in multiple cancer types which are rarely ALT-positive, that is, where *ATRX* loss is likely a random passenger event rather than central to oncogenesis (Supplementary Fig. S4B).

Further, expression levels for DRG2 have also been shown to be lower in ALT-positive cell lines compared to telomerase-positive cell lines [66], a finding we confirmed by comparing a panel of ALT-positive and ALT-negative cell lines (Supplementary Fig. S5A and B). Taken together, these findings implied a potential causal role for DRG2 loss in ALT-cancers. DRG2 is an evolutionarily conserved GTP-binding protein which plays important roles in cell growth/differentiation and tumorigenesis [67–70]. *DRG2* was reported to be significantly under expressed in *IDH1*-mutant astrocytoma, which are frequently *ATRX*-mutant/ALT-positive [71–73]. In our dataset, however, *IDH1* mutation status was not correlated with *DRG2* expression level, once *ATRX*-mutation status was also considered (Supplementary Fig. S5C).

Due to the strong association, *DRG2* was knocked down in ATRX-wildtype and ATRX-knockout HeLa LT cells (Fig. 4C). Depletion of DRG2 in ATRX-null cells led to a significant induction of ALT hallmarks (Fig. 4D–F). This was associated with a significant increase in markers of DNA damage, such as γH2AX and 53BP1 (Supplementary Fig. S5D–G).

Importantly, DRG2 has recently been associated with cellular and DNA responses to oxidative stress and regulation of mitochondrial metabolism, with depletion of the gene leading to mitochondrial dysfunction, elevated ROS levels and downregulation of antioxidant genes including *SOD1* and *SOD2* [74–76]. We found that knockdown of DRG2 led to a significant increase in cellular ROS levels in the HeLa LT cells (Fig. 4G). Furthermore, cellular ROS levels correlated with DRG2 expression in a panel of other ALT-positive cell lines (Fig. 4H). Given this, we assayed expression of a panel of antioxidant genes, focusing on those previously found to be dysregulated upon gene loss [69–71]. This analysis confirmed significant downregulation of numerous key ROS scavengers such as *SOD1*, *GPX1* and *SCARA3* (Fig. 4I). Crucially, the observed induction of ALT in ATRX-knockout HeLa LT cells was counteracted by co-treatment with the ROS-scavenger NAC, suggesting that the induction of the ALT pathway was indeed dependent on the presence of ROS (Fig. 4J–L).

### Exposure to hypoxia induces ALT in ATRX-deficient cells

**Figure 5.**
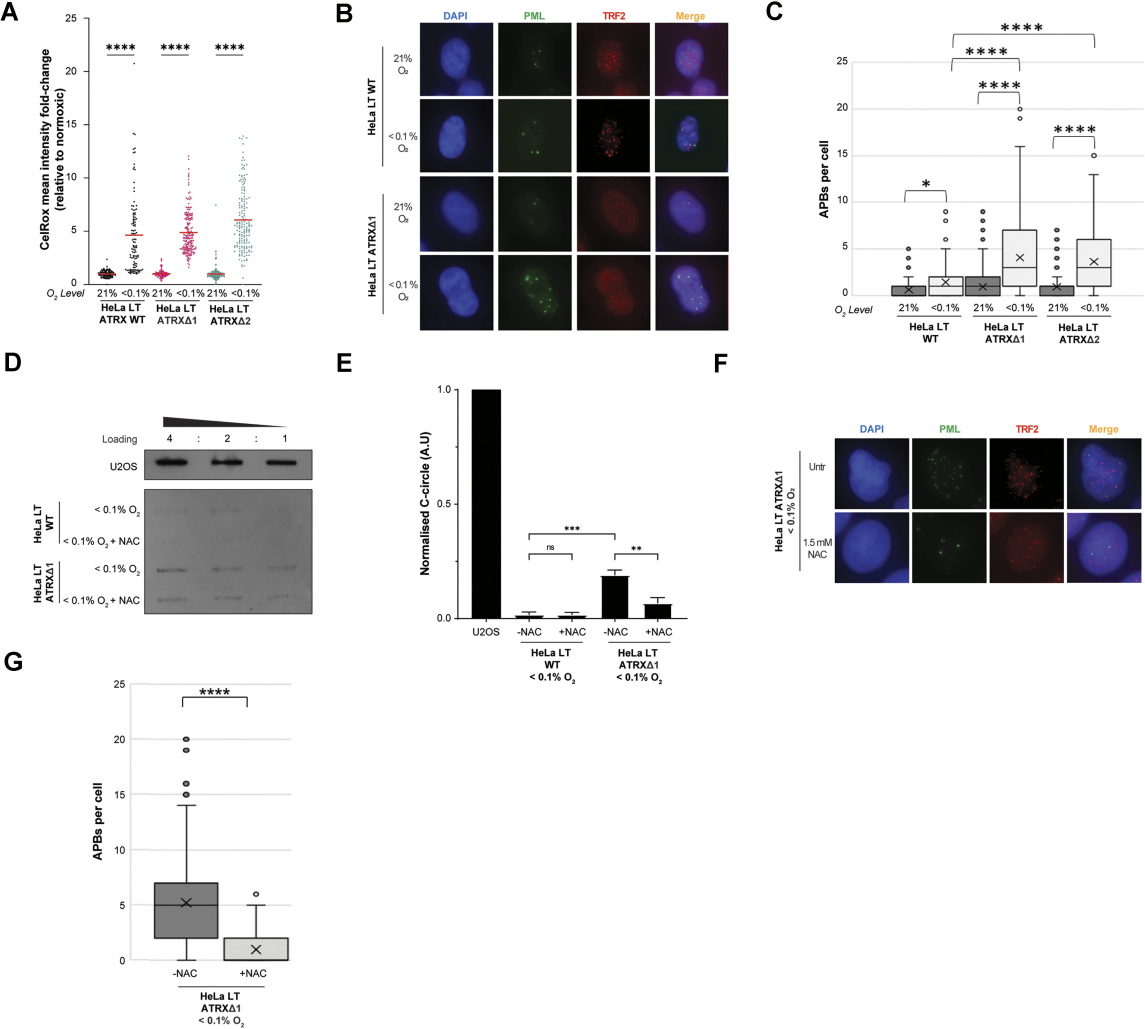
Exposure of ATRX-null cells to radiobiological hypoxia leads to ROS-mediated ALT induction. (**A**) CellRox assay showed that all cell lines had elevated ROS levels under radiobiological hypoxic conditions (<0.1% O_2_), two biological replicates. (**B**) Representative images showing that exposure to <0.1% O_2_ for 6 h led to induction of APBs in both ATRX-wildtype and ATRX-null HeLa LT cells, but the effect was much more marked in the latter. (**C**) Quantification of panel (B), >100 cells analysed for each condition, Kruskall–Wallis test. (**D**) Exposure to <0.1% O_2_ for 6 h, followed by 24 h of cell growth in normoxia, demonstrated induction of C-circles in ATRX-null but not ATRX-wildtype cells; the effect was reversed by concurrent treatment with NAC. (**E**) Quantification of panel (D), unpaired *t*-test, *n* = 2. (**F**) Representative images showing that the observed induction of APBs in ATRX-null HeLa LT cells was reduced by concurrent treatment with 1.5 mM NAC for 48 h. (**G**) Quantification of panel (E), >100 cells analysed for each condition, Kruskall–Wallis test.

While mutational loss of SETD2 and/or downregulation of DRG2 (and consequent dysregulation of redox genes) present two genetic routes by which natural ALT cells could have higher levels of ROS, we also considered other possible nongenetic factors. The tumour microenvironment is often hypoxic, and under these conditions there is accumulation of ROS [38, 77–79]. An assay for total cellular ROS levels (CellRox) confirmed elevated ROS levels in the tested cell lines exposed to radiobiological hypoxia (<0.1% O_2_) for 6 h, as well as with H_2_O_2_ treatment as a positive control (Fig. 5A and Supplementary Fig. S6A). This brief exposure to hypoxic conditions was sufficient for robust induction of APBs in HeLa LT cells lacking ATRX, with a much milder – albeit significant – effect on wildtype cells (Fig. 5B and C). While there did not appear to be any effect on C-circles in HeLa LT cells (*data not shown*), it was considered that the 6-h timeframe might be insufficient for *de novo* generation of C-circles; the cells were, therefore, exposed to radiobiological hypoxia for 6 h, followed by growth in normoxia for 24 h. Under these conditions, a modest but significant induction of C-circles was observed (Fig. 5D and E). The induction of ALT-markers by hypoxia was reduced by co-treatment with NAC (Fig. 5D–G).

Finally, the effect of hypoxic conditions on cell viability was assessed by clonogenic survival assay, demonstrating that HeLa LT cells lacking ATRX had a small but significant survival advantage as compared to ATRX-replete cells at 16 and 24 h timepoints (Supplementary Fig. S6B). This suggests that under extreme environmental conditions, such as hypoxia, the ability of cells to initiate the ALT pathway might confer a small survival advantage. This has previously been shown in two ALT-positive osteosarcoma cell lines, and was thought to be due to elevated levels of ARG2 [80].

### Excessive ROS promote ALT induction through the generation of R-loops

Data presented here strongly suggested that conditions of increased ROS resulted in initiation of the ALT pathway in ATRX-deplete cells. Excessive ROS levels have been shown to strongly induce R-loop formation at specific genomic loci, which, in turn, has been shown to recruit RAD52, and facilitate BIR-mediated repair of telomeric oxidative damage [81, 82]. It has also recently been shown that global increases in ROS levels can induce replication fork stalling that is associated with R-loop formation, while exposure to hypoxia has been demonstrated to lead to increased R-loop levels in a ROS-dependent manner [38, 83]. It was considered, therefore, whether the elevated ROS levels assayed herein resulted in R-loops and whether this might be causal of ALT-pathway activity.

**Figure 6.**
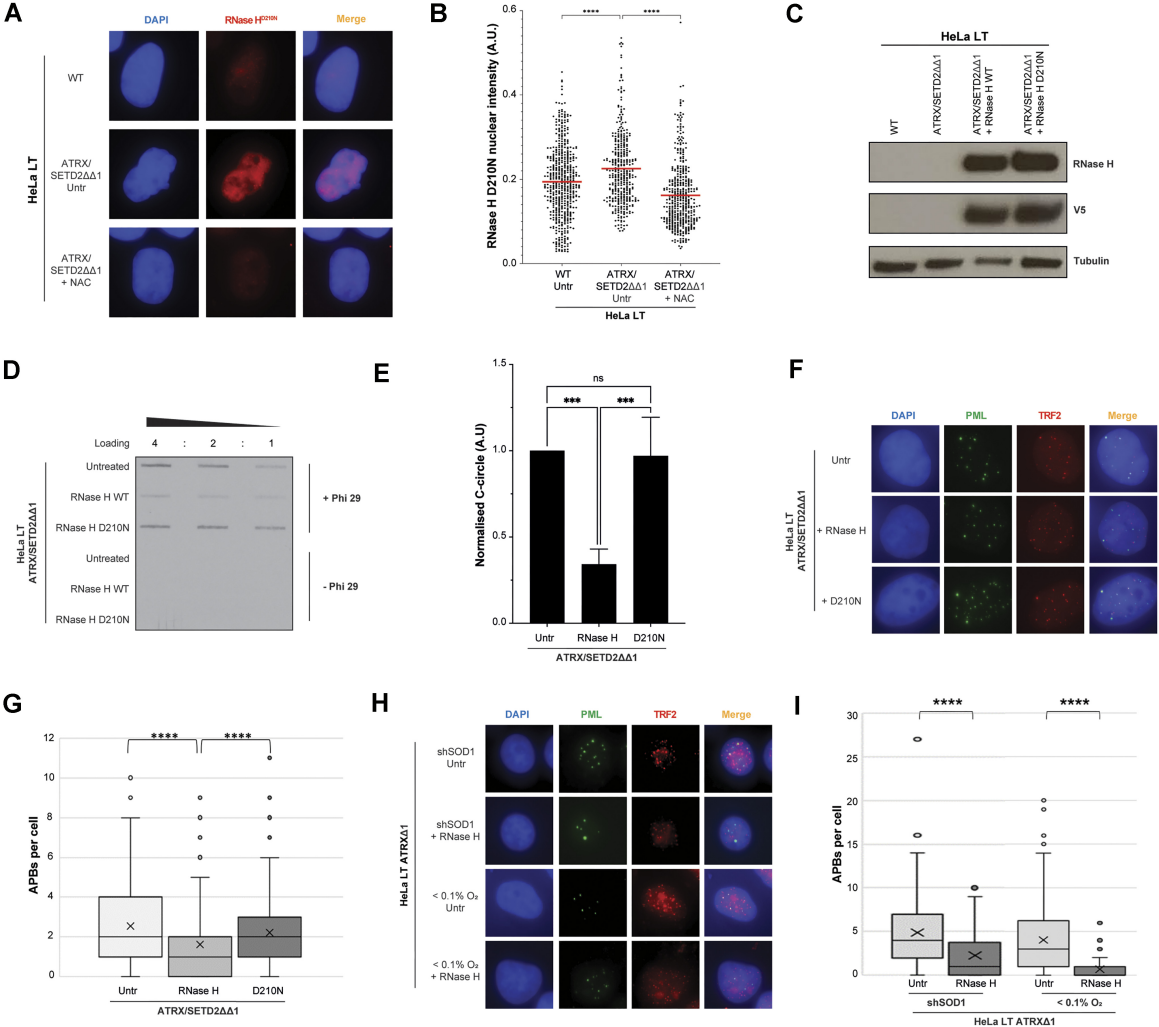
Elevated ROS generate R-loops, which drive ALT pathway activity. (**A**) Representative immunofluorescence images of indicated cell lines transduced with V5-tagged RNase H1^D210N^. Staining for V5 was carried out and nuclear intensity determined. The analysis demonstrated that ATRX/SETD2 double knockout cells had significantly higher global R-loop levels than the wildtype cells, but this increase was prevented by 1.5 mM NAC treatment. See the ‘Materials and methods’ section for details. (**B**) Quantification of panel (A), >100 cells analysed across each condition, unpaired *t*-test. (**C**) Immunoblot confirming overexpression of the V5-tagged RNaseH WT and D210N constructs. (**D**) Representative blot showing that overexpression of RNaseH led to a reduction in C-circle levels; this reduction was not observed upon overexpression of the D210N catalytically dead mutant. (**E**) Quantification of panel (D), unpaired *t*-test, *n* = 4. (**F**) Representative images showing that the induced production of APBs in ATRX/SETD2 double knockout cells is reduced by RNase H1 overexpression, but not the D210N mutant. (**G**) Quantification of panel (F), >100 cells analysed for each condition, Kruskall–Wallis test. (**H**) Representative images showing that the induced production of APBs in ATRX-null cells depleted of SOD1 or exposed to <0.1% O_2_ is significantly reduced by RNase H1 overexpression. (**I**) Quantification of panel (H), >100 cells analysed for each condition, Kruskall–Wallis test.

Immunoprecipitation using an antibody specific to RNA-DNA hybrids (S9.6) showed a marked accumulation of telomeric R-loops upon loss of SETD2 (Supplementary Fig. S7A and B). RNase H1 is an endonuclease enzyme which can cleave R-loops; pre-treatment with RNase H1 decreased the signal, confirming antibody specificity (Supplementary Fig. S7A and B). We further confirmed that generation of ROS was promoting R-loop formation by assessing total R-loop levels in the absence and presence of anti-oxidant therapy. We observed that upon treatment with NAC, the total nuclear level of R-loops significantly decreased (Fig. 6A and B). We next sought to determine whether the observed accumulation of telomeric R-loops was responsible for facilitating the accumulation of ALT markers in the combined ATRX/SETD2 knockout cells. RNase H1 overexpression in the cells resulted in a significant reduction of C-circles and APBs and this was not observed upon overexpression of the catalytically dead RNase H1 D210N mutant (Fig. 6C–G). Upon treatment with RNase H1, a reduction of APBs was also observed in cells treated with shSOD1 and in those exposed to hypoxia, two other sources of ROS that we have shown can trigger ALT activation in ATRX-null cells (Fig. 6H and I). Together, this suggests that ALT induction by elevated ROS is at least partially mediated via the formation of R-loops.

### ROS-mediated induction of ALT is associated with the formation of DPCs

We next considered whether or not the observations herein were consistent with our recent work, which demonstrated that formation of DPCs is a potent trigger for the initiation of ALT in ATRX-null cells [28]. Many enzymes – such as topoisomerases, polymerases, and poly(ADP-ribose) polymerases – form reversible intermediates with DNA during catalysis, and these intermediates might form covalent DPCs or tightly chromatin-bound noncovalent complexes [84]. Furthermore, exposure to ROS is a well-recognized cause of DPC formation [85, 86]. It was, therefore, considered whether the observed induction of ALT under various conditions of elevated ROS was related to formation of DPCs.

First, the levels of TOP1 covalent complexes (TOP1cc) in cells was assessed, as our previous work has demonstrated their importance in ALT pathway induction, although other proteins are also likely to be implicated [28]. Knockdown of *SOD1* caused a significant increase in TOP1cc in both *ATRX* wildtype and *ATRX* knockout cells; however, the effect was more striking in ATRX-deficient cells, indicating a possible synergistic effect in this combination (Supplementary Fig. S8A). There was also a significant increase in TOP1cc levels in ATRX-deficient, but not ATRX-replete HeLa LT cells when exposed to hypoxic conditions (Supplementary Fig. S8B). In SETD2 knockout cells, there was a striking increase in TOP1cc and the effect was additive upon combined ATRX/SETD2 loss (Supplementary Fig. S8C). Given that ROS levels do not increase further upon combined knock-out of ATRX and SETD2 (Fig. 2A), this additional accumulation of TOP1ccs cannot be explained by additional ROS-induced R-loops. It is plausible that the observed further increase could be directly explained by loss of ATRX, as this has been shown to result in accumulation of R-loops [22, 23]. In addition to genome wide TOP1cc, there was an increase in co-localization of TOP1cc at telomeres in all treatment conditions assayed (Supplementary Fig. S8D–F). Importantly, co-treatment with NAC reduced the accumulation of TOP1cc under all conditions tested (Supplementary Fig. S8G and H).

Given the particularly striking accumulation of TOP1cc following treatment with shSOD1, it was considered whether the observed induction of ALT was reliant on TOP1 trapping. Co-depletion of both SOD1 and TOP1 abolished the previously observed production of APBs in *ATRX*-deplete cells lines (Supplementary Fig. S9A–C). It was concluded, therefore, that generation of excessive ROS was leading to increased trapped proteins (such as TOP1cc) and that this, in turn, induced the ALT phenotype in *ATRX*-deplete cells. Interestingly, it was also observed that RNase H1 overexpression decreased the previously observed trapping of TOP1cc, implicating R-loop formation as causative of some TOP1cc formation (Supplementary Fig. S9D–F).

### Elevation of ROS levels can be used therapeutically in ALT-positive sarcoma and glioma

**Figure 7.**
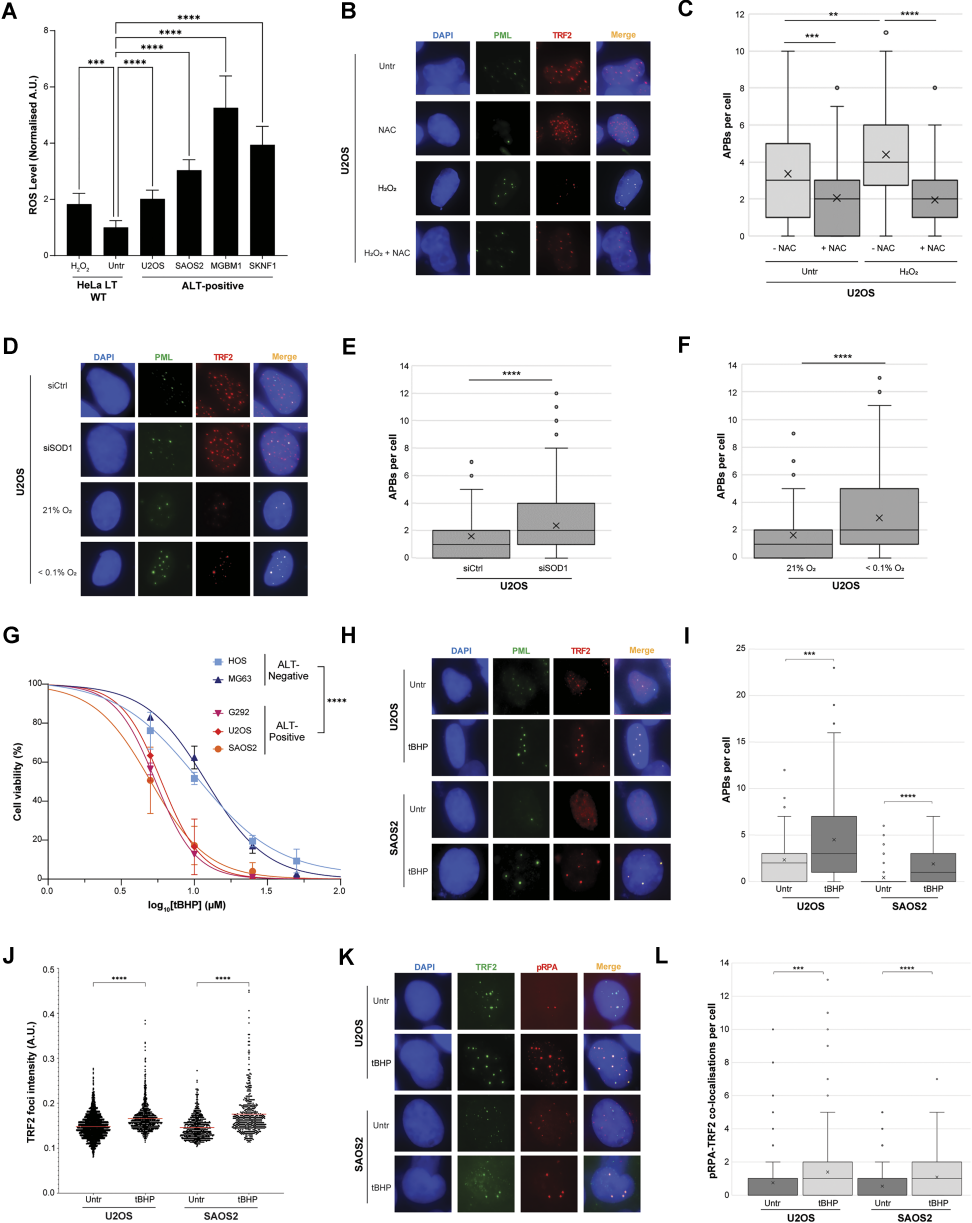
Therapeutic potential of modulating ROS levels in ALT-positive cancers. (**A**) Analysis of ROS levels by DCFDA assay demonstrated that ALT-positive cell lines had generally high levels of ROS, all at least equivalent to that observed in H_2_O_2_ treated HeLa LT wildtype (ALT-negative) control cells (2 biological replicates, 20 technical replicates). (**B**) Representative images showing that APB levels in U2OS cells are significantly elevated following 100 μM H_2_O_2_ treatment, while they are significantly reduced following 1.5 mM NAC treatment, both for 48 h. (**C**) Quantification of panel (B), >100 cells analysed for each condition, Kruskall–Wallis test. (**D**) Representative images showing that APB levels in U2OS cells are significantly elevated following SOD1 depletion or exposure to <0.1% O_2_. (**E**, **F**) Quantification of panel (D), >100 cells analysed for each condition, Kruskall–Wallis test. (**G**) A panel of ALT-positive and ALT-negative osteosarcoma cell lines were treated with tBHP at the indicated range of concentrations and left for 5 days. Live cells were counted using the CellTiter Glo luminescent cell viability assay. Data are presented as mean values ± standard deviation from three biological replicates. (**H**) Representative images showing an increase in APBs in U2OS and SAOS2 cells treated with 10 μM tBHP for 48 h. (**I**) Quantification of panel (H), >100 cells analysed for each condition, Kruskall–Wallis test. (**J**) Quantification of TRF2 foci intensity, showing a significant increase in U2OS and SAOS cells upon 10 μM tBHP treatment for 48 h, >100 cells analysed for each condition, unpaired *t*-test. (**K**) Representative images showing an increase in TRF2-pRPA-S33 co-localizations in U2OS and SAOS2 cells treated with 10 μM tBHP for 48 h. (**L**) Quantification of panel (K), >100 cells analysed for each condition, Kruskall–Wallis test.

The data from our *in vitro* system strongly suggested that elevated levels of ROS was a critical mechanism underpinning the evolution of ALT pathway in ATRX-deficient cells. We therefore explored whether there was evidence of elevated ROS levels in ALT-positive cell lines. Analysis of various ALT-positive cell lines demonstrated that they had high levels of ROS, similar to (or greater than) HeLa LT cells treated with 400 μM of H_2_O_2_ (Fig. 7A). The gene expression signature of TCGA samples was analysed for patterns associated with hypoxia using a gene signature for HIF-pathway activation, as described previously [87]. This demonstrated that ATRX-mutant LGG had a significantly higher hypoxia signature score, indicating higher levels of hypoxia in these tumours, which is known to be associated with elevated ROS [77–79] (Supplementary Fig. S10A). The same effect was not, however, observed in osteosarcoma or HGG, indicating that hypoxia might not be a root cause of elevated ROS in these lines, or that hypoxia plays a role in both ATRX-wildtype and ATRX-null tumours of this type (Supplementary Fig. S10B and C).

A key consideration is whether these insights into ALT-pathway biology can be used to guide targeted treatment strategies in ALT-positive cancers. Approaches which have aimed to reduce ALT-pathway activity have produced disappointing results and so, in recent years, approaches have shifted to focusing on methods which can induce ALT-pathway overactivity [19,20]. Excessive ALT-activity is associated with genetic instability and cell death – the so-called hyper-ALT phenotype [7, 88]. Exposure of the archetypal ALT-positive osteosarcoma cell line, U2OS, to H_2_O_2_, hypoxia or SOD1 gene silencing resulted in increased APBs, indicating increased ALT-pathway activity (Fig. 7B–F). Conversely, treatment with NAC significantly reduced ALT pathway markers (Fig. 7B and C and Supplementary Fig. S10D and E). Similarly, increased APBs were observed in two other ALT-positive cell lines – SAOS2 (osteosarcoma) and MGBM1 (HGG) – upon H_2_O_2_ treatment (Supplementary Fig. S10F and G).

We next tested the effect of tBHP treatment in a panel of osteosarcoma cell lines, due to its greater stability over H_2_O_2_ in cell culture. It was observed that ALT-positive osteosarcoma cells were exquisitely more sensitive to tBHP, as compared to ALT-negative osteosarcoma cell lines (Fig. 7G and Table 1). Treatment of ALT-positive sarcomas with tBHP resulted in markers of hyper-ALT, including increased APBs, increased telomere clustering/intensity and replication stress (Fig. 7H–L and Supplementary Fig. S11A and B).

**Table 1.** Sensitivity of a panel of osteosarcoma cell lines to tBHP

Cell line	IC_50_	95% C.I.	*P*-value
HOS	10.42	9.34–11.62	
MG63	12.07	11.26–12.94	
*Pooled ALT-negative*	*11.25*	*10.48–12.08*	*n/a*
U2OS	5.96	5.18–6.80	
SAOS2	5.04	3.94–5.88	
G292	5.43	5.33–5.54	
*Pooled ALT-positive*	*5.47*	*5.13–5.79*	*<.0001*

Analysis of cell viability demonstrated a markedly reduced IC_50_ in ALT-positive osteosarcoma cell lines as compared to ALT-negative counterparts.

Taken together, we conclude that an increase in ROS levels predisposes a cell to ALT activity, through increased R-loop and/or DPC formation – although the exact mechanism by which this occurs requires further investigation. Elevated levels of cellular ROS might result from specific ALT-associated mutations, gene dysregulation, and/or changes in the tumour microenvironment, such as hypoxia. In the context of ATRX loss, elevated ROS levels facilitate the initiation and maintenance of the ALT pathway, and this has the potential to be therapeutically manipulated.

## Discussion

The evolution of a telomere maintenance mechanism is a central event in the malignant transformation of a pre-cancerous cell. The ability of a cell to elongate shortened telomeres – thereby bypassing the usual safety mechanisms of cellular senescence and programmed cell death – is a prerequisite to limitless cell division and has, therefore, been defined as a hallmark of cancer. In a sizeable subset of cancers, telomere elongation occurs via the ALT pathway. Whilst it has now been established that loss of *ATRX* (or *DAXX*) is a central genetic event in the evolution of ALT, the field has been perplexed by the insufficiency of this change in isolation. Extensive research has failed to reveal a universal second genetic factor that defines ALT cancer biology: here, we present a model whereby the second factor is not, necessarily, genetic in nature, but an environmental pressure which provides the evolutionary niche for ATRX-null cells to flourish. Through a focus on glioma tumours, we have identified that generation of excessive ROS can act as a potent and essential driving factor in ATRX-deficient malignant cells. In particular, we have defined two novel sources of ROS in gliomas: co-mutation of *SETD2* in HGG and downregulation of *DRG2* in LGG.

**Figure 8.**
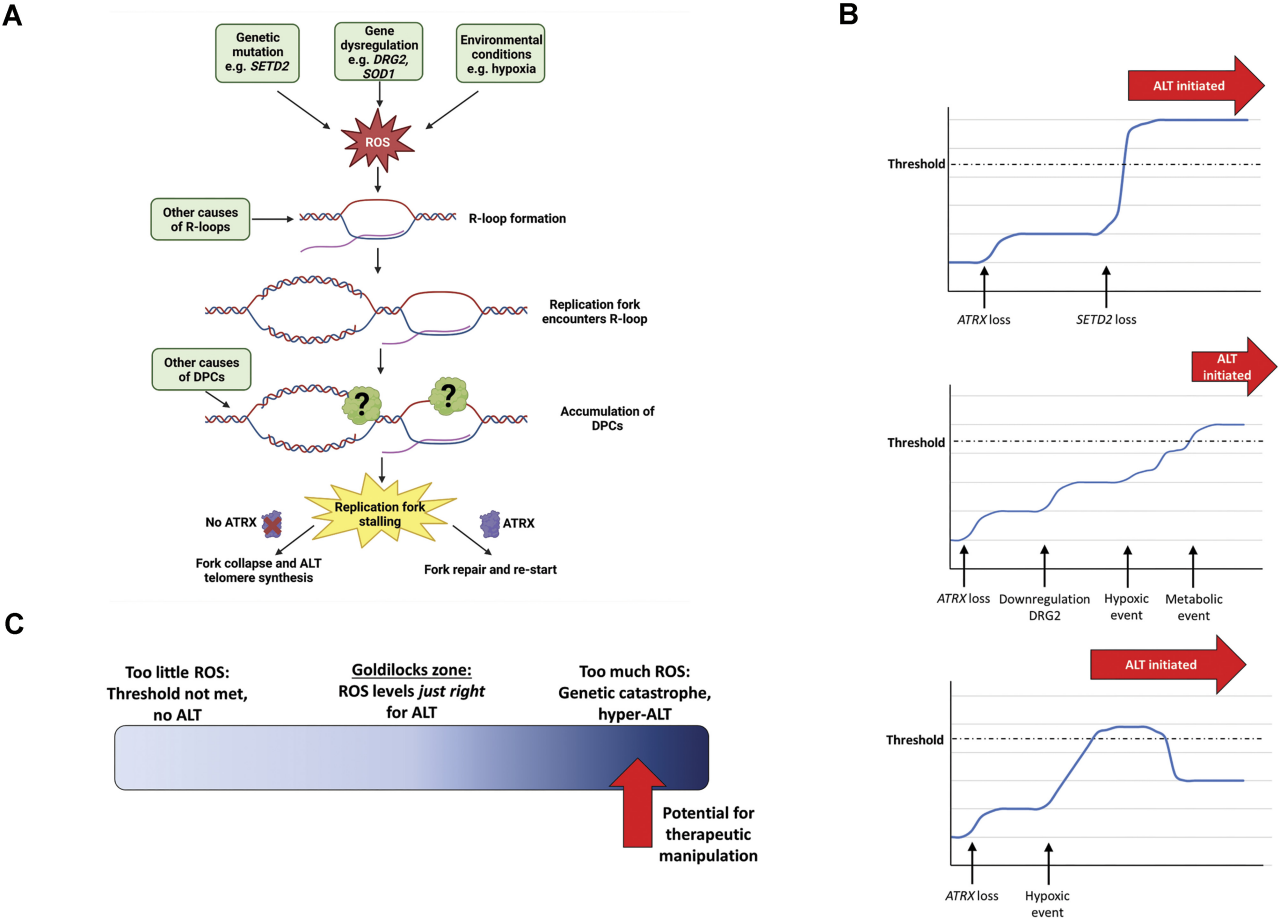
A novel model of ALT-pathway induction, highlighting interplay between genetic and environmental factors. (**A**) Evolutionary model of ALT cancers. The environmental causation of ALT telomere maintenance is elevated ROS levels, which can arise from genetic events (such as loss of SETD2 or downregulation of DRG2), tumour microenvironment events (such as hypoxia) or other cellular/metabolic events (such as inflammation or metabolic adaptation). Elevated ROS levels lead to formation of R-loops which, in turn, trap DNA-interacting proteins, such as TOP1, forming DPCs. R-loops and DPCs present a barrier to the DNA replication machinery, leading to replication fork stalling, which can be protected and/or restarted by ATRX. In the absence of ATRX, however, the fork cannot be re-started, leading to aberrant downstream processing and generation of telomeric DNA DSBs, which provide the substrate for telomere recombination and elongation. Loss of ATRX, therefore, can be considered the proximal (genetic) causation of ALT telomere maintenance. Created in BioRender. Goncalves, T. (2025) https://BioRender.com/u23t978 (**B**) ALT induction requires ATRX-deficient cells to reach a threshold of R-loops/DPCs, above which there is initiation of telomere recombination. ATRX loss alone causes a small elevation of R-loops, but this is insufficient to meet the threshold. Sometimes, a second genetic event – such as loss of SETD2 – causes a marked elevation above the threshold (upper panel). It is likely in most cases, however, that a cumulative effect of environmental factors will elevate the level above threshold (middle panel). It could be conjectured that a one-off event that generates a spike in ROS (such as hypoxia caused by thromboembolism) could reach the threshold, with subsequent drop below the threshold again (lower panel). However, as ALT is a self-perpetuating phenomenon, once the threshold has been reached, subsequent changes may not affect ALT activity. (**C**) ALT cancer cells exist in an optimal zone, where ROS levels are in fine balance between lack of ALT activity and genetic crisis. Therapeutic manipulation to elevate ROS levels and push cells into hyper-ALT phenotype presents a novel therapeutic strategy for ALT cancers.

Cancers occur due to the process of clonal evolution, which has traditionally been understood through a gene-centric model of driver mutations. This model does not, however, consider the complex interplay between stochastic mutational processes and natural selection through environmental factors [89]. A more comprehensive model of cancer takes consideration of the epigenetic/genetic changes (proximal causation), as well as the dynamic and context-specific environmental selection forces which allow clonal advantage (evolutionary causation). Here, we have demonstrated that for ALT-pathway activity in glioma (and possibly other ALT-cancer types), the proximal genetic causation is *ATRX* loss, whilst the evolutionary causation can be accumulation of ROS (Fig. 8A). ROS are produced by many cellular compartments, including phagosomes, endoplasmic reticulum, cell membrane, peroxisomes, and mitochondria [90].

Here, we have identified loss of *SETD2*, dysregulation of redox genes such as *DRG2* and exposure to hypoxia as sources of excessive ROS in ALT-positive gliomas. First, we identified that mutations in the histone methyltransferase *SETD2* could trigger ALT induction in ATRX-deficient cells. *SETD2* loss was associated with elevated ROS levels, due to the dysregulation of redox pathway genes. This agrees with recent research into inflammatory bowel disease, which found that SETD2 loss in the gut led to excessive ROS [55, 56]. Co-mutation of *SETD2* and *ATRX* occurs in up to 18% of paediatric HGG and, as such, this finding sheds light on the genetic aetiology of these highly aggressive tumours. It is important to note, however, that whilst co-treatment of ATRX/SETD2 knockout cells with anti-oxidants *reduced* the level of R-loops and ALT-pathway markers, it did not completely ablate them. This suggests that loss of SETD2 might be driving R-loop accumulation and ALT activity by more than one mechanism. Previous work has shown that disrupting the interaction between THO and the Sin3A histone deacetylase (HDAC) complex leads to increased R-loop levels [91]. SETD2 is the writer of the H3K36me3 mark, which is vital for HDAC recruitment to chromatin and, therefore, SETD2 loss has been speculated to increase R-loops through reduced HDAC recruitment [43]. Additionally, SETD2 has previously been shown to recruit the FACT (facilitates chromatin transcription) complex to actively transcribed regions [92]. The FACT complex has been shown to prevent R-loop accumulation, potentially by maintaining an open chromatin structure [93, 94]; therefore, one could speculate that SETD2 might restrict the formation of R-loops through promoting FACT recruitment.

Secondly, this work has extended knowledge on the relatively poorly understood gene, *DRG2*. We demonstrated a striking correlation between loss of the *ATRX* gene and downregulation of *DRG2* expression in tumour types which are frequently ALT-positive, including LGG and HGG. Importantly, tumour types which are not commonly ALT-positive (where *ATRX* mutation is a random passenger event) did not show a correlation between ATRX loss and decreased *DRG2* expression, supporting a causal role for DRG2 in ALT tumorigenesis.

It was observed that knockdown of the gene led to robust induction of ALT in cells lacking ATRX, but not in wildtype cells. Mechanistically, we found that decreased DRG2 loss was associated with reduced expression of key antioxidant genes – including *SCARA3*, *GPX1* and *SOD1*, with consequent elevation in cellular ROS levels – mirroring the findings of other recent research into this gene [67, 74, 75]. We also found that direct knockdown of *SOD1* was able to induce ALT-pathway activity in the ATRX-null cells. This implies that dysregulation of SOD1 and other redox genes is, at least in part, responsible for the observed induction of ALT-pathway activity upon loss of expression of *DRG2*.

We further demonstrated that exposure to hypoxia (with consequent generation of ROS) was also able to precipitate ALT-pathway induction in cells lacking ATRX, and we present evidence that ATRX-mutant LGG have gene signatures more strongly associated with hypoxia than ATRX-wildtype LGG tumours. It will be important to explore this link in more detail in both glioma cell lines and *in vivo*. For example, an magnetic resonance imaging-based study might be considered, to assess hypoxia in ATRX-deficient glioma. In addition to the three factors identified here, there are many other potential sources of cellular ROS – such as inflammation, mitochondrial DNA mutations, or metabolic adaptations [95] – which might all be contributory to the ALT phenotype in different tumours. Importantly, hypoxic conditions have recently been described to lead to an increase in R-loops in a ROS-dependent manner [38].

Elevated ROS levels underpin several key events in cancer initiation and progression, such as angiogenesis, cell signalling, and metastasis, but also cause changes to DNA structure. For example, oxidation of guanine by ROS leads to formation of 8-oxoG, one of the most prevalent altered bases in the genome. One recent study explored the effect of 8-oxoG at telomeres and found that targeted 8-oxoG formation at telomeres in ALT-positive cell lines stimulates ALT activity [96]. They demonstrated that telomeric 8-oxoG was a source of replication stress, as evidenced by fragile telomeres and other markers [96]. Another type of DNA conformational change caused by ROS is the formation of R-loops [82]. There is some evidence that presence of 8-oxoG can precipitate formation of R-loops, possibly through interference with normal R-loop enzymatic processing by RNase H1 [97, 98]. Persistent or excessive R-loops are a potent source of replication stress, DNA damage and genomic instability [99]. Recent work has shown that ROS lead to global slowing of replication fork velocity and this effect was found to be dependent on formation of co-transcriptional R-loops [83]. Given the role of ATRX in the protection and restart of stalled replication forks, this warrants further investigation [31, 100–102].

Further, formation of R-loops at telomeres – particularly those including TERRA sequence – have been shown to be pivotal in ALT telomere synthesis. It is well-recognized that ALT cancer cells harbour higher levels of R-loops and this is partially attributable to loss of ATRX. ATRX has a role in the regulation of R-loops, with loss of ATRX being associated with increased R-loop formation at telomeric sequences [22]. The observed accumulation of R-loops upon ATRX loss is not, however, sufficient to drive ALT in isolation, else ATRX loss alone would be sufficient to induce the ALT phenotype; hence, there must be other causes for R-loop accumulation. Here, we greatly extend our knowledge about the source of excessive R-loops in ALT cells by suggesting that when ATRX loss occurs in the context of elevated ROS, an R-loop threshold is reached and ALT telomere synthesis occurs (Fig. 8B). It has previously been demonstrated that, once initiated, ALT telomere synthesis is a self-perpetuating phenomenon [103]. It is possible, therefore, that after an event where the R-loop threshold is reached, ALT would persist following the resolution or discontinuation of that environmental pressure (Fig. 8B).

We recently demonstrated that formation of covalent DPCs, as well as noncovalent PARP-DNA complexes, in the absence of ATRX induced ALT-pathway activity [28]. It is well established that elevated ROS can lead to the accumulation of various DPCs [85, 86] and, as such, we explored the role of DPC formation in ATRX-deficient cells under conditions of elevated ROS. We confirmed that ROS did indeed cause an increase in TOP1cc, a DPC formed from a catalytic intermediate of TOP1. Further, conditions of elevated ROS (such as silencing of SOD1) were *not* able to induce ALT when there was concurrent silencing of TOP1, indicating that formation of TOP1cc was integral to ROS-mediated ALT-pathway induction. Curiously, accumulation of TOP1cc under conditions of elevated ROS was partially abrogated by overexpression of RNase H1, suggesting that the observed accumulation of DPCs was at least partially dependent on R-loops. There is some evidence of interplay between TOP1cc and R-loops, indicating shared resolution of these abnormal structures [104, 105]. Our data suggest that R-loops are a key contributor to the formation of DPCs under conditions of elevated ROS, but the exact mechanism by which this occurs needs to be explored in future work. R-loops are a major source for impairment of DNA replication forks and so can contribute to transcription-replication conflicts, where the replication machinery is slowed (or stalled) by a transcription event. It is possible, then that the slowed replication bubbles are more prone to form DPCs, but it is also possible that DPCs form directly on R-loops. These options will each need to be investigated by careful structural and biochemical assays.

Here, we demonstrated that cells lacking ATRX had a modest survival advantage over ATRX-wildtype cells in hypoxic environments. This observation is consistent within our proposed model, where somatic clonal evolution selects for the subclones with the stochastic genetic changes most fitted to environmental pressures. We also observed, however, that when ROS levels were further elevated – such as through silencing of SOD1 or treatment with H_2_O_2_ – ATRX-null clones were less able to tolerate these conditions, and were selectively vulnerable. This observation highlights the fine-balance required in ROS homeostasis in cancer cells. It could be considered that there is an optimal level of ROS for cells lacking ATRX: too little ROS, and the threshold of R-loops/DPCs is not met, and ALT telomere synthesis is not triggered. Further elevation of ROS levels, however, leads to excessive ALT-pathway activity, increased DNA damage and ultimately cell death – the hyper-ALT phenotype – as ATRX-null cells have intrinsic vulnerability to DNA damage due to the many roles of ATRX in genome integrity. There is, however, a level at which ROS are ‘*just right*’, where ATRX-deficient cells can leverage ROS-induced R-loops (and subsequent DPC formation) to allow telomere maintenance and limitless cell division (Fig. 8C). Therapeutic manipulation of ROS levels to outside of the optimal level provides a novel approach to treatment for ATRX-deficient ALT cancers, including osteosarcoma, LGG and other ALT-positive tumours.

Our data strongly supports the hypothesis that therapeutic elevation of ROS could be used to induce hyper-ALT. We demonstrated that treatment of natural ALT-positive cell lines – including glioma and osteosarcoma – led to hyper-ALT, with concurrent elevated DNA damage markers and cellular sensitivity. It will be important to further explore the therapeutic usage of ROS in the treatment of these cancer types, through development of new drug agents and study of microtissues and animal models. Finally, given our observation that increased ROS led to accumulation of DPCs, the synergy between ROS-generating therapies and DPC-generating agents should be explored. Outcomes for people affected by ALT-positive cancers remain very poor, and translational advances in therapeutic approaches must remain the most urgent priority for research in the field.

## Supplementary Material

gkaf061_Supplemental_File

## Data Availability

The RNA-seq data from low grade glioma is available through The Cancer Genome Atlas (https://portal.gdc.cancer.gov/). Data used to generate the volcano plot is available at https://doi.org/10.5281/zenodo.14678092. All other data is included within the main text or supplemental files of the manuscript.
