# Supplementary material for: Elevated reactive oxygen species can drive the alternative lengthening of telomeres pathway in ATRX-null cancers
Source: Nucleic Acids Res. 2025 Feb 8;53(4):gkaf061. doi: 10.1093/nar/gkaf061 (PMC11806356; doi:10.1093/nar/gkaf061)
Supplement: gkaf061_Supplemental_File [file gkaf061_supplemental_file.pdf]

Elevated reactive oxygen species can drive the Alternative Lengthening of Telomeres pathway in ATRX-null cancers

Tomas Goncalves,[1] Siobhan Cunniffe,[2] Tiffany S. Ma,[2] Natalie Mattis,[3] Andrew W. Rose,[4] Thomas Kent,[1] David R. Mole,[5] Helene E.B. Geiller,[2] Linda van Bijsterveldt,[2] Timothy C. Humphrey,[2] Ester M. Hammond,[2] Richard J. Gibbons,[1] David Clynes,[2*] and Anna M. Rose[1,3*]

[1] MRC Molecular Haematology Unit, Weatherall Institute of Molecular Medicine, University of Oxford, Oxford, OX3 9DS

[2] Department of Oncology, University of Oxford, Oxford, OX3 7DQ

[3] Department of Paediatrics, University of Oxford, Oxford, OX3 9DU

[4] Department of Physics, Faculty of Natural Sciences, Imperial College, London, SW7 2BW

[5] Nuffield Department of Medicine, University of Oxford, Oxford, OX3 7BN

* To whom correspondence should be addressed. Tel: 01865 234234 Email: anna.rose@paediatrics.ox.ac.uk

Correspondence may also be addressed to: david.clynes@oncology.ox.ac.uk

SI Contents

Table S1 – TaqMan array assay of an antioxidant gene panel *Page 2*

Table S2 – Primer sequences used *Page 7*

Supplementary Figures *Page 8*

Table S1 - TaqMan array assay of an antioxidant gene panel

Cell Line	Gene	Rep 1	Rep 2	Rep 3	Average	Std Dev
HeLa LT WT	GAPDH	1.0000	1.0000	1.0000	1.0000	0.0000
HeLa LT ATRXΔ1		1.0000	1.0000	1.0000	1.0000	0.0000
HeLa LT SETD2Δ1		1.0000	1.0000	1.0000	1.0000	0.0000
HeLa LT WT	ALB-Hs00910225_m1	1.0000	1.0000	1.0000	1.0000	0.0000
HeLa LT ATRXΔ1		n/a	5.5899	3.0066	n/a	n/a
HeLa LT SETD2Δ1		2.2494	14.4792	4.0805	6.9364	6.5961
HeLa LT WT	ALOX12-Hs00167524_m1	n/a	n/a	n/a	n/a	n/a
HeLa LT ATRXΔ1		n/a	n/a	n/a	n/a	n/a
HeLa LT SETD2Δ1		n/a	n/a	n/a	n/a	n/a
HeLa LT WT	ANGPTL7-Hs00221727_m1	n/a	n/a	n/a	n/a	n/a
HeLa LT ATRXΔ1		n/a	n/a	n/a	n/a	n/a
HeLa LT SETD2Δ1		n/a	n/a	n/a	n/a	n/a
HeLa LT WT	AOX1-Hs00154079_m1	1.0000	1.0000	1.0000	1.0000	0.0000
HeLa LT ATRXΔ1		1.2227	1.2321	1.4420	1.2990	0.1240
HeLa LT SETD2Δ1		3.3705	5.7787	2.1704	3.7732	1.8375
HeLa LT WT	APOE-Hs00171168_m1	1.0000	1.0000	1.0000	1.0000	0.0000
HeLa LT ATRXΔ1		0.1647	0.5035	0.1952	0.2878	0.1874
HeLa LT SETD2Δ1		0.5486	1.1865	0.4912	0.7421	0.3859
HeLa LT WT	ATOX1-Hs00187841_m1	1.0000	1.0000	1.0000	1.0000	0.0000
HeLa LT ATRXΔ1		0.7991	1.8004	0.5045	1.0347	0.6793
HeLa LT SETD2Δ1		0.9307	1.0896	0.8641	0.9615	0.1159
HeLa LT WT	BNIP3-Hs00969291_m1	1.0000	1.0000	1.0000	1.0000	0.0000
HeLa LT ATRXΔ1		0.5448	0.3857	0.5288	0.4864	0.0876
HeLa LT SETD2Δ1		0.6265	0.2103	0.4707	0.4358	0.2103
HeLa LT WT	CAT-Hs00156308_m1	1.0000	1.0000	1.0000	1.0000	0.0000
HeLa LT ATRXΔ1		0.7840	1.2690	0.7691	0.9407	0.2844
HeLa LT SETD2Δ1		0.5627	1.0458	0.7253	0.7779	0.2458
HeLa LT WT	CCL5-Hs00174575_m1	1.0000	1.0000	1.0000	1.0000	0.0000
HeLa LT ATRXΔ1		0.0960	0.4237	0.1436	0.2211	0.1771
HeLa LT SETD2Δ1		2.9352	3.7481	1.4573	2.7135	1.1614
HeLa LT WT	CCS-Hs00192851_m1	1.0000	1.0000	1.0000	1.0000	0.0000
HeLa LT ATRXΔ1		0.6692	1.3362	0.9220	0.9758	0.3367
HeLa LT SETD2Δ1		0.5953	1.0850	0.8094	0.8299	0.2455
HeLa LT WT	CSDE1-Hs00918650_m1	1.0000	1.0000	1.0000	1.0000	0.0000
HeLa LT ATRXΔ1		1.1853	1.4309	0.8816	1.1659	0.2751
HeLa LT SETD2Δ1		0.8405	1.1992	0.7076	0.9158	0.2543
HeLa LT WT	CYBA-Hs03044361_m1	1.0000	1.0000	1.0000	1.0000	0.0000
HeLa LT ATRXΔ1		0.9160	1.3141	0.7645	0.9982	0.2838
HeLa LT SETD2Δ1		1.3112	1.2292	1.1910	1.2438	0.0614
HeLa LT WT	CYGB-Hs00370478_m1	1.0000	n/a	1.0000	n/a	n/a
HeLa LT ATRXΔ1		n/a	n/a	0.7230	n/a	n/a
HeLa LT SETD2Δ1		5.5892	n/a	0.9812	n/a	n/a
HeLa LT WT	DGKK-Hs01385661_m1	n/a	n/a	n/a	n/a	n/a
HeLa LT ATRXΔ1		n/a	n/a	n/a	n/a	n/a
HeLa LT SETD2Δ1		n/a	n/a	n/a	n/a	n/a
HeLa LT WT	DHCR24-Hs00207388_m1	1.0000	1.0000	1.0000	1.0000	0.0000
HeLa LT ATRXΔ1		0.9624	1.5418	0.9090	1.1377	0.3509
HeLa LT SETD2Δ1		1.1684	1.8204	1.3327	1.4405	0.3391

HeLa LT WT	DUOX1-Hs00213694_m1	n/a	n/a	n/a	n/a	n/a
HeLa LT ATRXΔ1		n/a	n/a	n/a	n/a	n/a
HeLa LT SETD2Δ1		n/a	n/a	n/a	n/a	n/a
HeLa LT WT	DUOX2-Hs00204187_m1	1.0000	1.0000	1.0000	1.0000	0.0000
HeLa LT ATRXΔ1		38.4793	5.3213	0.7510	14.8505	20.5903
HeLa LT SETD2Δ1		150.8259	25.5248	4.7252	60.3587	79.0342
HeLa LT WT	DUSP1-Hs00610256_g1	1.0000	1.0000	1.0000	1.0000	0.0000
HeLa LT ATRXΔ1		0.1525	1.2051	0.4553	0.6043	0.5418
HeLa LT SETD2Δ1		0.2048	1.0610	0.9019	0.7226	0.4554
HeLa LT WT	EPHX2-Hs00157403_m1	1.0000	1.0000	1.0000	1.0000	0.0000
HeLa LT ATRXΔ1		0.7501	1.3221	0.5555	0.8759	0.3985
HeLa LT SETD2Δ1		0.8806	2.0185	1.4421	1.4471	0.5689
HeLa LT WT	EPX-Hs00946094_m1	n/a	n/a	n/a	n/a	n/a
HeLa LT ATRXΔ1		n/a	n/a	n/a	n/a	n/a
HeLa LT SETD2Δ1		n/a	n/a	n/a	n/a	n/a
HeLa LT WT	FOXM1-Hs01073586_m1	1.0000	1.0000	1.0000	1.0000	0.0000
HeLa LT ATRXΔ1		0.9078	1.4212	0.8266	1.0519	0.3224
HeLa LT SETD2Δ1		0.5892	1.0439	0.7964	0.8098	0.2277
HeLa LT WT	GLRX2-Hs00375015_m1	1.0000	1.0000	1.0000	1.0000	0.0000
HeLa LT ATRXΔ1		1.1262	1.4627	0.9690	1.1860	0.2522
HeLa LT SETD2Δ1		0.7411	1.0012	0.8596	0.8673	0.1302
HeLa LT WT	GPR156-Hs00537796_m1	1.0000	1.0000	1.0000	1.0000	0.0000
HeLa LT ATRXΔ1		0.5672	1.0674	0.7209	0.7852	0.2562
HeLa LT SETD2Δ1		0.5539	0.7379	0.6354	0.6424	0.0922
HeLa LT WT	GPX1-Hs00829989_gH	1.0000	1.0000	1.0000	1.0000	0.0000
HeLa LT ATRXΔ1		1.2279	1.5019	0.7192	1.1497	0.3972
HeLa LT SETD2Δ1		0.4427	0.8603	0.7240	0.6757	0.2130
HeLa LT WT	GPX2-Hs01591589_m1	1.0000	1.0000	1.0000	1.0000	0.0000
HeLa LT ATRXΔ1		32.8321	5.5425	2.7633	13.7126	16.6162
HeLa LT SETD2Δ1		20.4208	3.7567	0.9918	8.3898	10.5105
HeLa LT WT	GPX3-Hs00173566_m1	n/a	n/a	n/a	n/a	n/a
HeLa LT ATRXΔ1		n/a	n/a	n/a	n/a	n/a
HeLa LT SETD2Δ1		n/a	n/a	n/a	n/a	n/a
HeLa LT WT	GPX4-Hs00989766_g1	1.0000	1.0000	1.0000	1.0000	0.0000
HeLa LT ATRXΔ1		0.8442	1.2887	0.7670	0.9666	0.2816
HeLa LT SETD2Δ1		0.7562	1.1232	0.6799	0.8531	0.2370
HeLa LT WT	GPX5-Hs00559733_m1	n/a	n/a	n/a	n/a	n/a
HeLa LT ATRXΔ1		n/a	n/a	n/a	n/a	n/a
HeLa LT SETD2Δ1		n/a	n/a	n/a	n/a	n/a
HeLa LT WT	GPX6-Hs00699698_m1	n/a	n/a	n/a	n/a	n/a
HeLa LT ATRXΔ1		n/a	n/a	n/a	n/a	n/a
HeLa LT SETD2Δ1		n/a	n/a	n/a	n/a	n/a
HeLa LT WT	GPX7-Hs00210410_m1	1.0000	1.0000	1.0000	1.0000	0.0000
HeLa LT ATRXΔ1		0.4153	0.5613	0.1281	0.3682	0.2204
HeLa LT SETD2Δ1		0.2513	0.1507	0.1739	0.1920	0.0527
HeLa LT WT	GSR-Hs00167317_m1	1.0000	1.0000	1.0000	1.0000	0.0000
HeLa LT ATRXΔ1		0.7703	1.3213	0.7378	0.9431	0.3279
HeLa LT SETD2Δ1		0.5479	1.1288	0.7119	0.7962	0.2994
HeLa LT WT	GSS-Hs00609286_m1	1.0000	1.0000	1.0000	1.0000	0.0000
HeLa LT ATRXΔ1		0.7839	1.6109	1.0409	1.1452	0.4233
HeLa LT SETD2Δ1		0.4554	1.1430	0.7355	0.7780	0.3458

HeLa LT WT	GSTZ1-Hs01041668_m1	1.0000	1.0000	1.0000	1.0000	0.0000
HeLa LT ATRXΔ1		0.7927	1.0962	0.8589	0.9159	0.1596
HeLa LT SETD2Δ1		0.5578	0.8478	0.6582	0.6879	0.1473
HeLa LT WT	GTF2I-Hs00263393_m1	1.0000	1.0000	1.0000	1.0000	0.0000
HeLa LT ATRXΔ1		0.9888	1.2479	0.8198	1.0188	0.2156
HeLa LT SETD2Δ1		1.0316	1.2181	0.8319	1.0272	0.1931
HeLa LT WT	KRT1-Hs00196158_m1	n/a	n/a	n/a	n/a	n/a
HeLa LT ATRXΔ1		n/a	n/a	n/a	n/a	n/a
HeLa LT SETD2Δ1		n/a	n/a	n/a	n/a	n/a
HeLa LT WT	LPO-Hs00413414_m1	n/a	n/a	n/a	n/a	n/a
HeLa LT ATRXΔ1		n/a	n/a	n/a	n/a	n/a
HeLa LT SETD2Δ1		n/a	n/a	n/a	n/a	n/a
HeLa LT WT	MBL2-Hs00175093_m1	n/a	n/a	n/a	n/a	n/a
HeLa LT ATRXΔ1		n/a	n/a	n/a	n/a	n/a
HeLa LT SETD2Δ1		n/a	n/a	n/a	n/a	n/a
HeLa LT WT	MGST3-Hs01058946_m1	1.0000	1.0000	1.0000	1.0000	0.0000
HeLa LT ATRXΔ1		0.7040	1.3591	0.5702	0.8778	0.4222
HeLa LT SETD2Δ1		0.5279	1.1227	0.7380	0.7962	0.3016
HeLa LT WT	MPO-Hs00924296_m1	n/a	n/a	1.0000	n/a	n/a
HeLa LT ATRXΔ1		n/a	n/a	n/a	n/a	n/a
HeLa LT SETD2Δ1		n/a	n/a	1.0632	n/a	n/a
HeLa LT WT	MPV17-Hs00159232_m1	1.0000	1.0000	1.0000	1.0000	0.0000
HeLa LT ATRXΔ1		0.6108	1.1464	0.8895	0.8822	0.2678
HeLa LT SETD2Δ1		0.5497	0.7747	0.5509	0.6251	0.1296
HeLa LT WT	MSRA-Hs00737166_m1	1.0000	1.0000	1.0000	1.0000	0.0000
HeLa LT ATRXΔ1		1.2184	1.5427	0.8683	1.2098	0.3373
HeLa LT SETD2Δ1		0.6147	0.5490	0.4882	0.5507	0.0633
HeLa LT WT	MT3-Hs01921768_s1	1.0000	1.0000	1.0000	1.0000	0.0000
HeLa LT ATRXΔ1		n/a	1.3812	0.7517	n/a	n/a
HeLa LT SETD2Δ1		n/a	1.0600	1.0756	n/a	n/a
HeLa LT WT	MTL5-Hs01127481_m1	1.0000	1.0000	1.0000	1.0000	0.0000
HeLa LT ATRXΔ1		1.0347	0.8757	0.4989	0.8031	0.2752
HeLa LT SETD2Δ1		2.4574	2.9293	1.3137	2.2335	0.8307
HeLa LT WT	NCF1-Hs00417167_m1	1.0000	1.0000	1.0000	1.0000	0.0000
HeLa LT ATRXΔ1		1.5906	1.3303	0.7139	1.2116	0.4502
HeLa LT SETD2Δ1		2.9891	5.3534	8.1264	5.4896	2.5714
HeLa LT WT	NCF2-Hs01084940_m1	1.0000	1.0000	1.0000	1.0000	0.0000
HeLa LT ATRXΔ1		3.6660	2.4000	1.6932	2.5864	0.9996
HeLa LT SETD2Δ1		37.2060	8.7766	3.0434	16.3420	18.2947
HeLa LT WT	NME5-Hs00177499_m1	1.0000	1.0000	1.0000	1.0000	0.0000
HeLa LT ATRXΔ1		n/a	n/a	0.7517	n/a	n/a
HeLa LT SETD2Δ1		51.1490	6.0535	4.2607	20.4877	26.5685
HeLa LT WT	NOS2-Hs01075529_m1	n/a	n/a	1.0000	n/a	n/a
HeLa LT ATRXΔ1		n/a	n/a	3.1034	n/a	n/a
HeLa LT SETD2Δ1		n/a	n/a	4.1956	n/a	n/a
HeLa LT WT	NOX5-Hs00225846_m1	1.0000	1.0000	1.0000	1.0000	0.0000
HeLa LT ATRXΔ1		1.2279	1.1681	0.8549	1.0836	0.2003
HeLa LT SETD2Δ1		10.0951	6.7397	5.6655	7.5001	2.3106
HeLa LT WT	NUDT1-Hs00159343_m1	1.0000	1.0000	1.0000	1.0000	0.0000
HeLa LT ATRXΔ1		0.9994	1.0443	0.8391	0.9610	0.1079
HeLa LT SETD2Δ1		0.7617	0.8658	0.7806	0.8027	0.0555

HeLa LT WT	OXR1-Hs00250562_m1	1.0000	1.0000	1.0000	1.0000	0.0000
HeLa LT ATRXΔ1		1.0485	0.9687	0.6849	0.9007	0.1911
HeLa LT SETD2Δ1		1.3068	0.9260	1.2229	1.1519	0.2000
HeLa LT WT	OXSR1-Hs00178247_m1	1.0000	1.0000	1.0000	1.0000	0.0000
HeLa LT ATRXΔ1		0.5143	1.4266	0.7311	0.8907	0.4766
HeLa LT SETD2Δ1		0.3875	0.9786	0.6676	0.6779	0.2957
HeLa LT WT	PDLIM1-Hs00253222_m1	1.0000	1.0000	1.0000	1.0000	0.0000
HeLa LT ATRXΔ1		0.5848	0.7072	0.3656	0.5525	0.1731
HeLa LT SETD2Δ1		1.3319	1.1506	0.7172	1.0666	0.3159
HeLa LT WT	PIP3-E-Hs00611486_m1	1.0000	1.0000	1.0000	1.0000	0.0000
HeLa LT ATRXΔ1		7.5046	1.3303	0.7517	3.1955	3.7430
HeLa LT SETD2Δ1		33.2374	12.7203	12.3155	19.4244	11.9641
HeLa LT WT	PNKP-Hs00892544_m1	1.0000	1.0000	1.0000	1.0000	0.0000
HeLa LT ATRXΔ1		0.9461	1.5914	1.1338	1.2238	0.3319
HeLa LT SETD2Δ1		0.6674	1.1652	1.0021	0.9449	0.2538
HeLa LT WT	PRDX1-Hs00602020_mH	1.0000	1.0000	1.0000	1.0000	0.0000
HeLa LT ATRXΔ1		0.9463	1.4785	0.7815	1.0687	0.3643
HeLa LT SETD2Δ1		0.6747	1.1047	0.7956	0.8584	0.2217
HeLa LT WT	PRDX2-Hs03044902_g1	1.0000	1.0000	1.0000	1.0000	0.0000
HeLa LT ATRXΔ1		0.9881	1.1517	1.0111	1.0503	0.0886
HeLa LT SETD2Δ1		1.1292	1.0542	1.1586	1.1140	0.0539
HeLa LT WT	PRDX3-Hs00428953_g1	1.0000	1.0000	1.0000	1.0000	0.0000
HeLa LT ATRXΔ1		0.9760	1.3820	0.9637	1.1073	0.2381
HeLa LT SETD2Δ1		0.6428	0.7648	0.6753	0.6943	0.0632
HeLa LT WT	PRDX4-Hs00197394_m1	1.0000	1.0000	1.0000	1.0000	0.0000
HeLa LT ATRXΔ1		2.5515	1.0224	0.8467	1.4735	0.9377
HeLa LT SETD2Δ1		1.9538	0.5621	0.7391	1.0850	0.7576
HeLa LT WT	PRDX5-Hs00738905_g1	1.0000	1.0000	1.0000	1.0000	0.0000
HeLa LT ATRXΔ1		0.7269	1.3898	0.8064	0.9744	0.3620
HeLa LT SETD2Δ1		0.6512	1.2735	0.9442	0.9563	0.3113
HeLa LT WT	PRDX6-Hs00705355_s1	1.0000	1.0000	1.0000	1.0000	0.0000
HeLa LT ATRXΔ1		0.8606	1.4812	0.9003	1.0807	0.3474
HeLa LT SETD2Δ1		0.5927	1.1457	0.9365	0.8916	0.2792
HeLa LT WT	PREX1-Hs00368207_m1	1.0000	1.0000	1.0000	1.0000	0.0000
HeLa LT ATRXΔ1		0.5024	1.4159	1.0817	1.0000	0.4622
HeLa LT SETD2Δ1		0.3643	0.9354	0.5850	0.6283	0.2880
HeLa LT WT	PRG3-Hs00196082_m1	n/a	n/a	1.0000	n/a	n/a
HeLa LT ATRXΔ1		n/a	n/a	n/a	n/a	n/a
HeLa LT SETD2Δ1		n/a	n/a	0.9720	n/a	n/a
HeLa LT WT	PRNP-Hs01920617_s1	1.0000	1.0000	1.0000	1.0000	0.0000
HeLa LT ATRXΔ1		0.4165	1.0814	0.4939	0.6639	0.3636
HeLa LT SETD2Δ1		1.7099	1.5455	1.4634	1.5729	0.1255
HeLa LT WT	PTGS1-Hs00377726_m1	1.0000	1.0000	1.0000	1.0000	0.0000
HeLa LT ATRXΔ1		0.1452	0.6760	0.3488	0.3900	0.2678
HeLa LT SETD2Δ1		0.0808	0.0769	0.1727	0.1101	0.0542
HeLa LT WT	PTGS2-Hs00153133_m1	1.0000	1.0000	1.0000	1.0000	0.0000
HeLa LT ATRXΔ1		0.1886	0.9237	0.4365	0.5163	0.3740
HeLa LT SETD2Δ1		1.6802	2.7253	2.5293	2.3116	0.5555
HeLa LT WT	PXDN-Hs00395488_m1	1.0000	1.0000	1.0000	1.0000	0.0000
HeLa LT ATRXΔ1		0.5581	0.9901	0.6711	0.7398	0.2240
HeLa LT SETD2Δ1		0.6550	0.9456	0.9707	0.8571	0.1755

HeLa LT WT	PXDNL-Hs00332687_m1	1.0000	1.0000	1.0000	1.0000	0.0000
HeLa LT ATRXΔ1		n/a	n/a	3.0066	n/a	n/a
HeLa LT SETD2Δ1		6.4287	3.3168	4.0805	4.6087	1.6218
HeLa LT WT	RNF7-Hs02621493_s1	1.0000	1.0000	1.0000	1.0000	0.0000
HeLa LT ATRXΔ1		0.4253	1.2196	0.5895	0.7448	0.4193
HeLa LT SETD2Δ1		0.2579	0.6731	0.8034	0.5781	0.2849
HeLa LT WT	SCARA3-Hs00939871_m1	1.0000	1.0000	1.0000	1.0000	0.0000
HeLa LT ATRXΔ1		0.3963	0.6853	0.5170	0.5329	0.1452
HeLa LT SETD2Δ1		0.2093	0.3393	0.1752	0.2413	0.0866
HeLa LT WT	SELS-Hs00218369_m1	1.0000	1.0000	1.0000	1.0000	0.0000
HeLa LT ATRXΔ1		1.1485	1.0635	0.6806	0.9642	0.2493
HeLa LT SETD2Δ1		1.2635	0.9936	0.9820	1.0797	0.1593
HeLa LT WT	SEPP1-Hs01032845_m1	1.0000	1.0000	1.0000	1.0000	0.0000
HeLa LT ATRXΔ1		2.1588	9.2435	2.1959	4.5328	4.0797
HeLa LT SETD2Δ1		63.8995	177.2598	117.8913	119.6835	56.7014
HeLa LT WT	SFTPD-Hs00358340_m1	n/a	n/a	1.0000	n/a	n/a
HeLa LT ATRXΔ1		n/a	n/a	0.7517	n/a	n/a
HeLa LT SETD2Δ1		n/a	n/a	3.4253	n/a	n/a
HeLa LT WT	SGK2-Hs00367639_m1	1.0000	1.0000	1.0000	1.0000	0.0000
HeLa LT ATRXΔ1		1.9170	1.2812	0.7517	1.3166	0.5835
HeLa LT SETD2Δ1		1.1153	3.8427	4.0347	2.9975	1.6329
HeLa LT WT	SIRT2-Hs00247263_m1	1.0000	1.0000	1.0000	1.0000	0.0000
HeLa LT ATRXΔ1		3.8659	1.5564	0.6700	2.0307	1.6499
HeLa LT SETD2Δ1		5.0559	1.3746	1.5154	2.6486	2.0860
HeLa LT WT	SOD1-Hs00533490_m1	1.0000	1.0000	1.0000	1.0000	0.0000
HeLa LT ATRXΔ1		0.9218	1.1961	0.6935	0.9371	0.2517
HeLa LT SETD2Δ1		0.3213	0.7692	0.4999	0.5302	0.2255
HeLa LT WT	SOD2-Hs00167309_m1	1.0000	1.0000	1.0000	1.0000	0.0000
HeLa LT ATRXΔ1		0.5378	0.8370	0.4674	0.6141	0.1963
HeLa LT SETD2Δ1		1.6851	1.2289	1.2642	1.3927	0.2538
HeLa LT WT	SOD3-Hs00162090_m1	1.0000	n/a	n/a	n/a	n/a
HeLa LT ATRXΔ1		n/a	n/a	n/a	n/a	n/a
HeLa LT SETD2Δ1		40.3997	n/a	n/a	n/a	n/a
HeLa LT WT	SRXN1-Hs00607800_m1	1.0000	1.0000	1.0000	1.0000	0.0000
HeLa LT ATRXΔ1		0.5152	1.0821	0.5981	0.7318	0.3062
HeLa LT SETD2Δ1		0.6124	1.0608	0.9305	0.8679	0.2307
HeLa LT WT	STK25-Hs01110460_m1	1.0000	1.0000	1.0000	1.0000	0.0000
HeLa LT ATRXΔ1		0.8925	1.2320	0.8765	1.0003	0.2008
HeLa LT SETD2Δ1		0.5473	0.8606	0.7883	0.7321	0.1641
HeLa LT WT	TPO-Hs00892519_m1	n/a	n/a	n/a	n/a	n/a
HeLa LT ATRXΔ1		n/a	n/a	n/a	n/a	n/a
HeLa LT SETD2Δ1		n/a	n/a	n/a	n/a	n/a
HeLa LT WT	TTN-Hs00399225_m1	n/a	n/a	n/a	n/a	n/a
HeLa LT ATRXΔ1		n/a	n/a	n/a	n/a	n/a
HeLa LT SETD2Δ1		n/a	n/a	n/a	n/a	n/a
HeLa LT WT	TXNDC2-Hs03405463_m1	n/a	n/a	n/a	n/a	n/a
HeLa LT ATRXΔ1		n/a	n/a	n/a	n/a	n/a
HeLa LT SETD2Δ1		n/a	n/a	n/a	n/a	n/a
HeLa LT WT	TXNRD1-Hs00917067_m1	1.0000	1.0000	1.0000	1.0000	0.0000
HeLa LT ATRXΔ1		0.4165	1.1518	0.4384	0.6689	0.4184
HeLa LT SETD2Δ1		0.5058	1.6259	0.6338	0.9219	0.6131

HeLa LT WT	TXNRD2-Hs00272352_m1	1.0000	1.0000	1.0000	1.0000	0.0000
HeLa LT ATRXΔ1		1.4120	1.7312	1.5013	1.5482	0.1647
HeLa LT SETD2Δ1		0.6330	0.8414	0.7149	0.7298	0.1050

Table S2 – Primer sequences used

Name	Sequence	Supplier/Origin
Beta-globin Fw	CGGCGGCGGGCGGCGCGGGCTGGGCGGCTTCATCCACGTTCACCTTG	Life Technologies
Beta-globin Rv	GCCCGGCCCGCCGCGCCCGTCCCGCCGGAGGAGAAGTCTGCCGTT	Life Technologies
HTeloC	TGTTAGGTATCCCTATCCCTATCCCTATCCCTATCCCTAACA	Life Technologies
HTeloG	ACACTAAGGTTTGGGTTTGGGTTTGGGTTTGGGTTAGTGT	Life Technologies
7SK Fw	GAGGGCGATCTGGCTGCGACAT	Life Technologies
7SK Rv	ACATGGAGCGGTGAGGGAGGAA	Life Technologies
DRG2 Fw	AGAGGACAGAGGCCAGACTT	Life Technologies
DRG2 Rv	ACCAGGGCGTACTTGAACTG	Life Technologies
GPX1 Fw	TATCGAGAATGTGGCGTCCC	Life Technologies
GPX1 Rv	TCTTGGCGTTCTCCTGAATGC	Life Technologies
SCARA3 Fw	GAATTGCAGGGAAGACAGGG	Life Technologies
SCARA3 Rv	GTAGAAGCTCTGGCTTCCTGG	Life Technologies
SOD1 Fw	ACTGGTGGTCCATGAAAAAGC	Life Technologies
SOD1 Rv	AACGACTTCCAGCGTTTCCT	Life Technologies

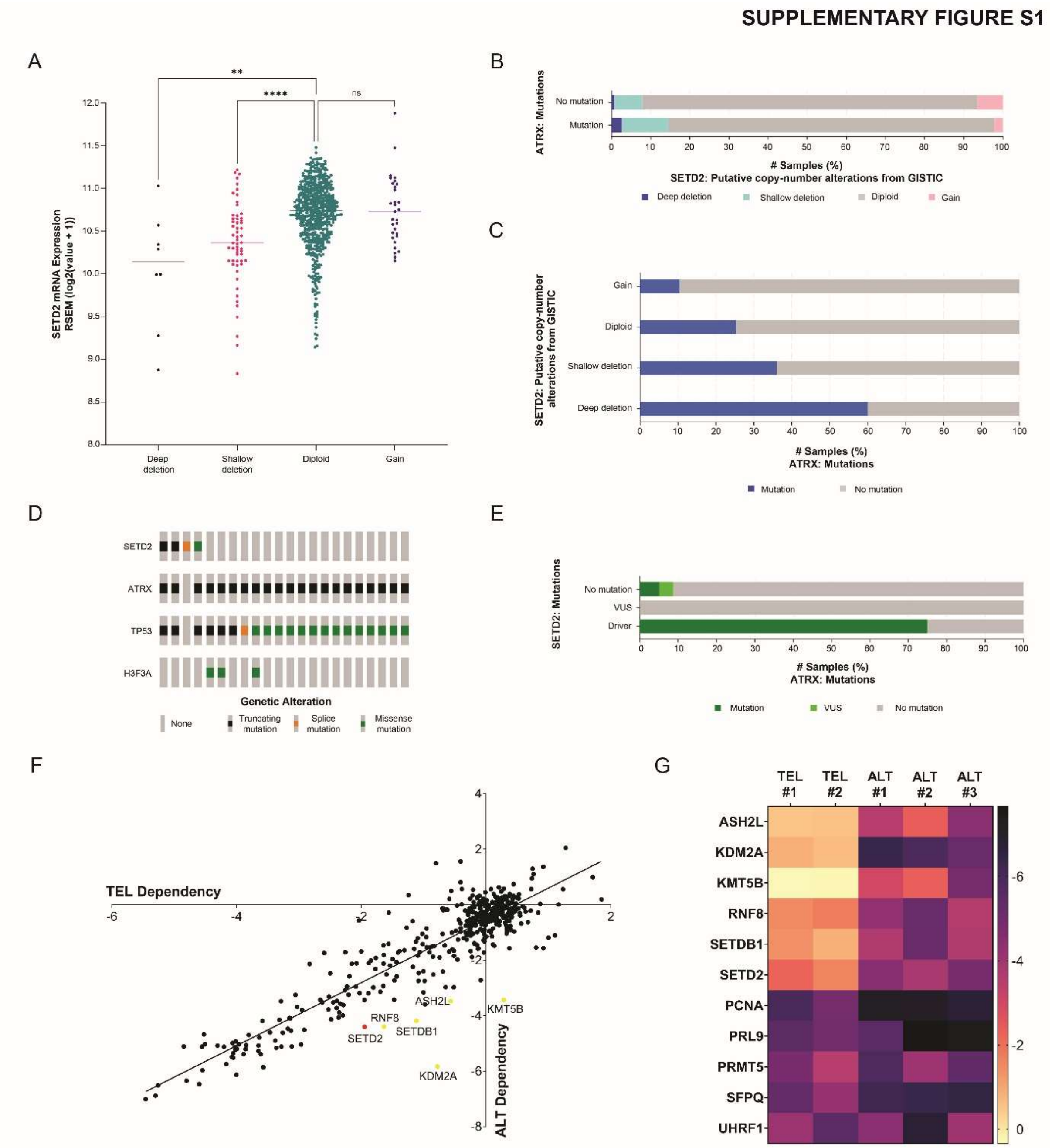

Figure S1: **Bioinformatics analysis of *SETD2* alterations in *ATRX* mutant glioma TCGA datasets.** (**A**) SETD2 copy-number variations (CNV), predicted from the GISTIC algorithm (*x-axis*) plotted against log2-transformed mRNA expression RSEM (batch normalized from Illumina hiseq_rnaseqv2) (*y-axis*). The GISTIC algorithm characterises CNVs as either deep deletions (homozygous), or shallow deletions (heterozygous). Mann-Whitney test. (**B**) 100% stacked bar graph showing percentage of samples containing CNV alterations in SETD2 against *ATRX* mutation status. (**C**) 100% stacked bar graph showing percentage of samples with *ATRX* mutations against SETD2 CNV alterations. (**D**) Schematic showing mutation status of *SETD2*, *ATRX*, *TP53* and *H3F3A* in the TCGA-HGG dataset. Note only those samples with *SETD2* and/or *ATRX* driver mutations are shown. (**E**) 100% stacked bar graph showing percentage of samples with *ATRX* mutations against *SETD2* mutations. VUS = variant of uncertain significance. (**F**) Gene dependency scores in an isogenic pair of ALT-negative (*x-*

axis) versus ALT-positive (*y-axis*) IMR90-T cells, generated with data from ref. (49). (**G**) Heatmap depicting log2 fold change of average sgRNA abundance of the indicated genes in cells after 16 population doublings, generated with data from ref. (49). Data in panels (A) – (C) is from the TCGA glioma datasets and data in panels (D) – (E) is from the TCGA-HGG dataset, accessed through cBioPortal. Data in panels (F) – (G) is from ref. (49).

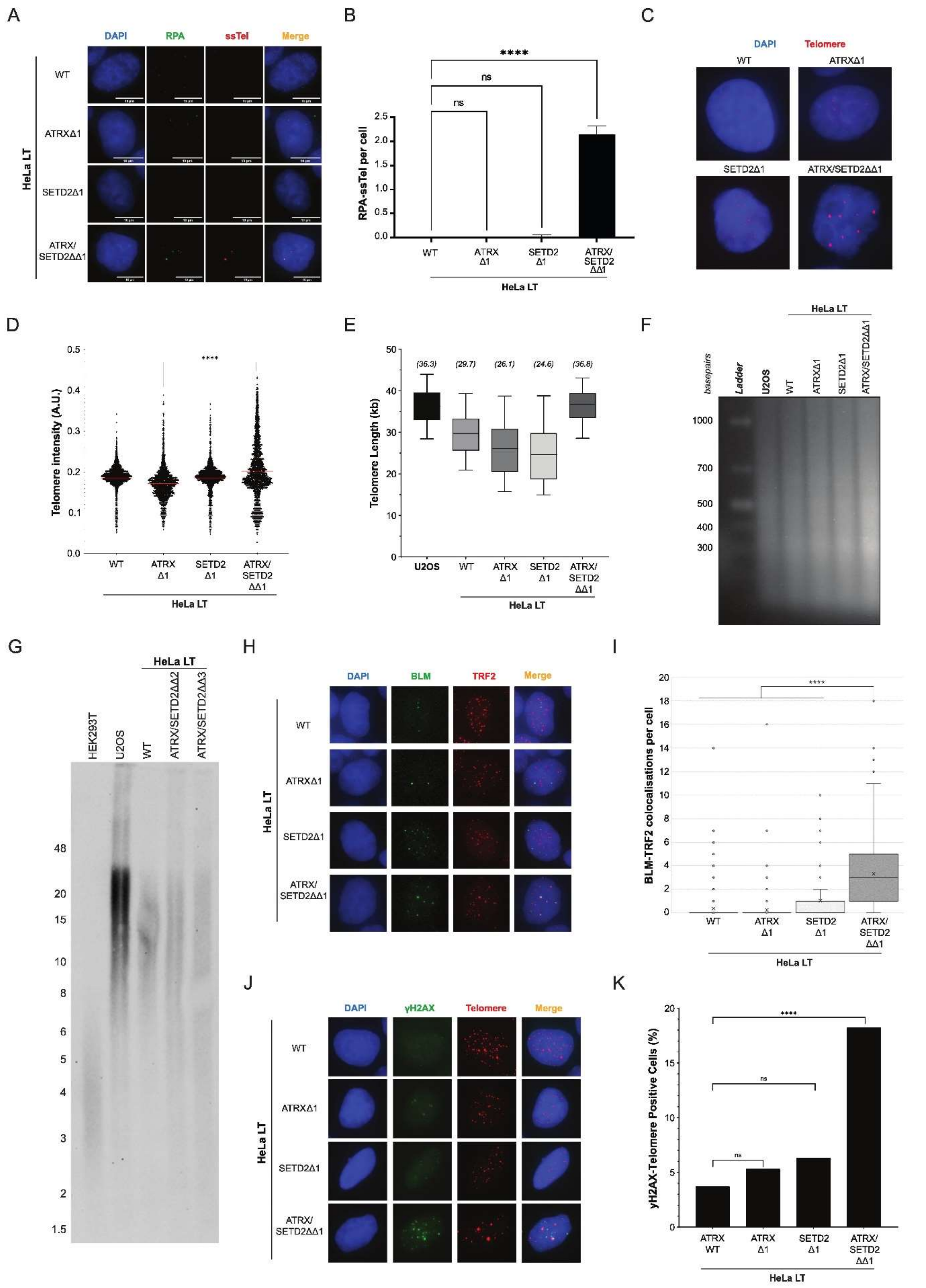

Figure S2: Concurrent loss of SETD2 and ATRX induces further markers of the ALT pathway activity. (**A**) Representative images showing that combined knockout of ATRX and

SETD2 induced RPA-ssTEL foci. (**B**) Quantification of (A), > 100 cells analysed for each condition, unpaired t-test. (**C**) Representative FISH images showing that combined ATRX/SETD2 loss leads to large, ultrabright telomeric foci. (**D**) Quantification of (C), > 100 cells analysed for each condition, unpaired t-test. (**E**) Quantification of the TRF-based Southern blot assay using the WALTER online toolset to estimate the median and interquartile range (36). Median values are displayed in parentheses above each bar. (**F**) Ethidium bromide stained gel from the TRF assay experiment, showing complete digestion of the genomic DNA (smear below ≈ 1000 bp) and equal loading. (**G**) Representative TRF-based Southern blot assay, showing telomere length distribution in the indicated cell lines. (**H**) Representative immunofluorescence images showing co-localisation between BLM and TRF2 upon loss of ATRX and SETD2. (**I**) Quantification of (G), > 100 cells analysed for each cell line, Kruskall-Wallis test. (**J**) Representative immunoFISH images showing co-localisation between γH2AX and telomeres upon loss of ATRX and SETD2. (**K**) Quantification of (I), > 100 cells analysed for each cell line, Chi-squared test.

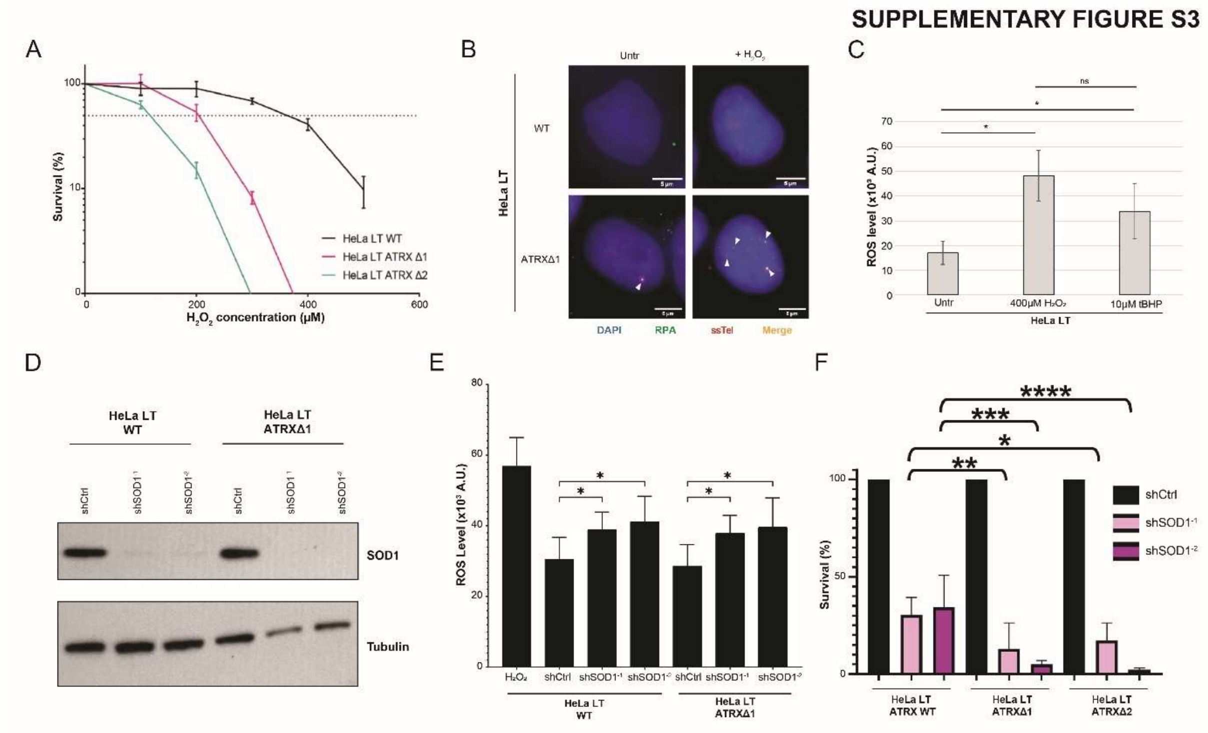

Figure S3: ATRX-null cells are more sensitive to elevated ROS levels. (**A**) Clonogenic survival assay demonstrated that ATRX-deficient cells were more sensitive to treatment with H_2O_2 than ATRX-wildtype cells. Three biological replicates, each run in triplicate. (**B**) Representative immunoFISH images showing co-localisation between RPA protein and ssTel in ATRX-null cells treated with H_2O_2. (**C**) DCFDA assay demonstrated elevated levels of ROS in HeLa LT cells treated with either 400 µM of H2O2 or 10 µM of t-BHP, unpaired t-test, 10 technical replicates. (**D**) Immunoblot demonstrating effective knockdown of SOD1 using shRNA. Tubulin was used as a loading control. (**E**) DCFDA assay demonstrated elevated levels of ROS in SOD1 knockdown cells, unpaired t-test, 10 technical replicates. (**F**) Celltiter Glo assay showed that ATRX-deficient cells displayed increased sensitiviy to shSOD1 as compared to ATRX-WT cells, 3 biological replicates, unpaired t-test.

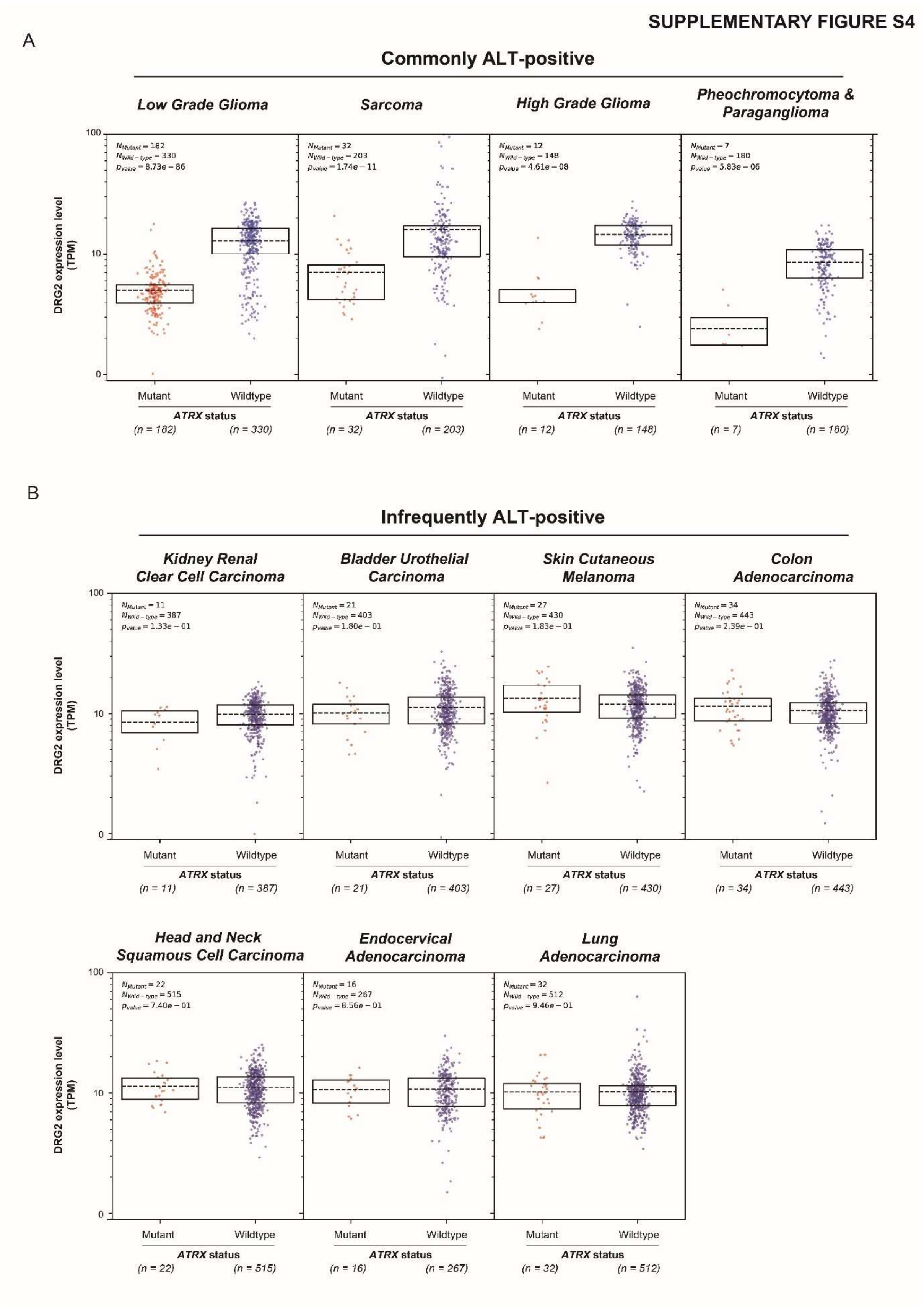
SUPPLEMENTARY FIGURE S4
A
Commonly ALT-positive
Low Grade Glioma
Sarcoma
High Grade Glioma
Pheochromocytoma & Paraganglioma
$N_{Mutant} = 182$, $N_{Wild-type} = 330$, $p_{value} = 8.73e-86$
$N_{Mutant} = 32$, $N_{Wild-type} = 203$, $p_{value} = 1.74e-11$
$N_{Mutant} = 12$, $N_{Wild-type} = 148$, $p_{value} = 4.61e-08$
$N_{Mutant} = 7$, $N_{Wild-type} = 180$, $p_{value} = 5.83e-06$
DRG2 expression level (TPM)
100
10
0
Mutant
Wildtype
ATRX status
(n = 182) (n = 330)
(n = 32) (n = 203)
(n = 12) (n = 148)
(n = 7) (n = 180)
B
Infrequently ALT-positive
Kidney Renal Clear Cell Carcinoma
Bladder Urothelial Carcinoma
Skin Cutaneous Melanoma
Colon Adenocarcinoma
$N_{Mutant} = 11$, $N_{Wild-type} = 387$, $p_{value} = 1.33e-01$
$N_{Mutant} = 21$, $N_{Wild-type} = 403$, $p_{value} = 1.80e-01$
$N_{Mutant} = 27$, $N_{Wild-type} = 430$, $p_{value} = 1.83e-01$
$N_{Mutant} = 34$, $N_{Wild-type} = 443$, $p_{value} = 2.39e-01$
(n = 11) (n = 387)
(n = 21) (n = 403)
(n = 27) (n = 430)
(n = 34) (n = 443)
Head and Neck Squamous Cell Carcinoma
Endocervical Adenocarcinoma
Lung Adenocarcinoma
$N_{Mutant} = 22$, $N_{Wild-type} = 515$, $p_{value} = 7.40e-01$
$N_{Mutant} = 16$, $N_{Wild-type} = 267$, $p_{value} = 8.56e-01$
$N_{Mutant} = 32$, $N_{Wild-type} = 512$, $p_{value} = 9.46e-01$
(n = 22) (n = 515)
(n = 16) (n = 267)
(n = 32) (n = 512)

Figure S4: Downregulation of *DRG2* in TCGA datasets of cancers that are frequently ALT-positive. Gene expression data, as transcripts per million (TPM), for selected TCGA cancer-type datasets that are commonly ALT-positive (**A**) and infrequently ALT-positive (**B**), showing expression of DRG2 in *ATRX*-mutant (red) versus *ATRX*-wildtype (blue).

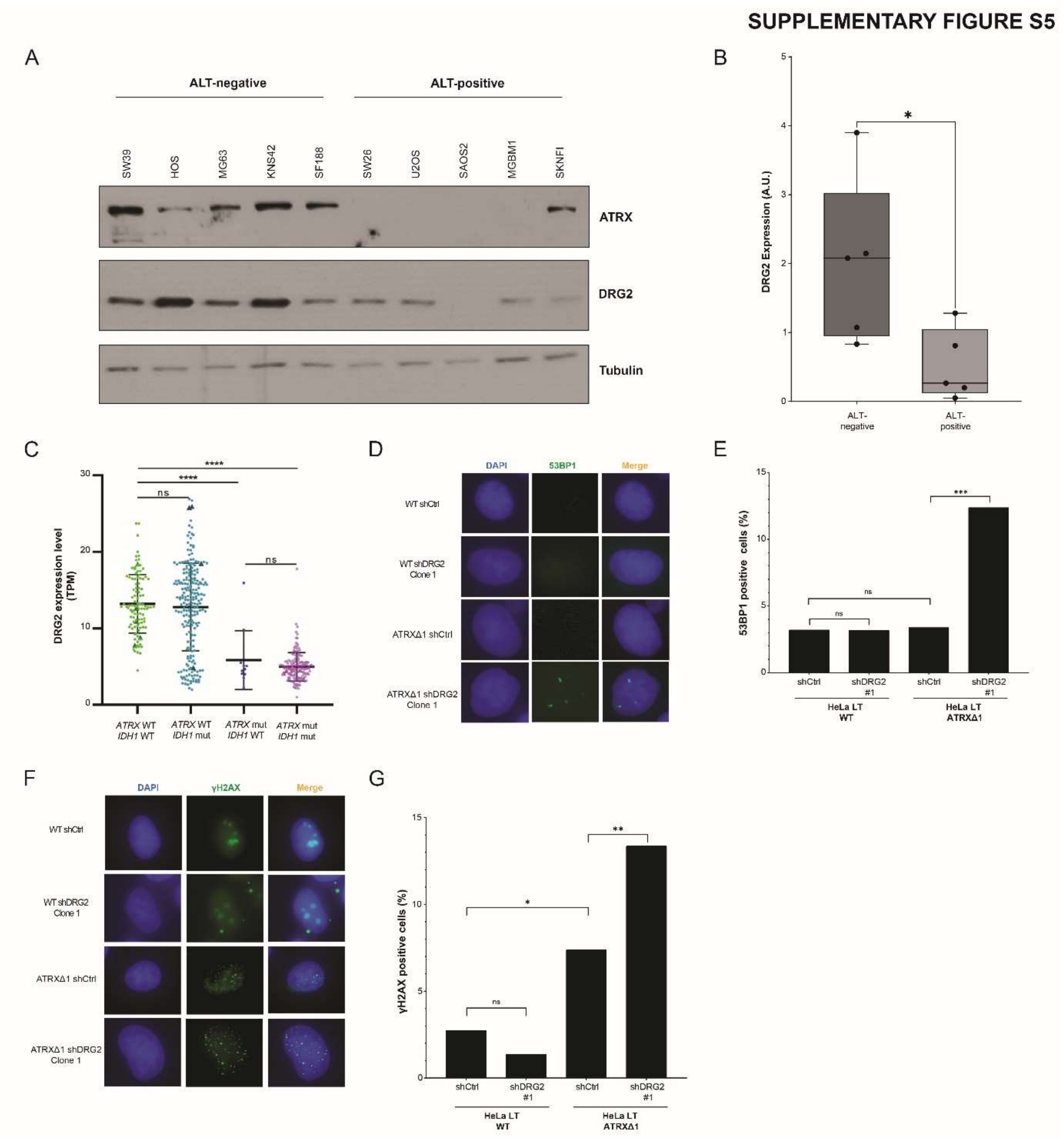

Figure S5: Downregulation of *DRG2* in ALT-positive cell lines. (**A**) Representative immunoblot of DRG2 levels in a panel of ALT-positive versus ALT-negative cell lines. (**B**) Quantification of (A), Mann-Whitney test, n=3. (**C**) *ATRX*-mutant low-grade glioma were found to have significantly lower expression of DRG2 as compared to *ATRX*-wildtype tumours and this did not correlate with IDH1 mutation status. (**D**) Representative immunofluorescence images showing elevated levels of 53BP1 foci in DRG2-depleted ATRX-null cells. (**E**) Quantification of (D), > 100 cells analysed for each condition, Chi-squared test. (**F**) Representative immunofluorescence images showing elevated levels of γH2AX foci in DRG2-depleted ATRX-null cells. (**G**) Quantification of (F), > 100 cells analysed for each condition, Chi-squared test.

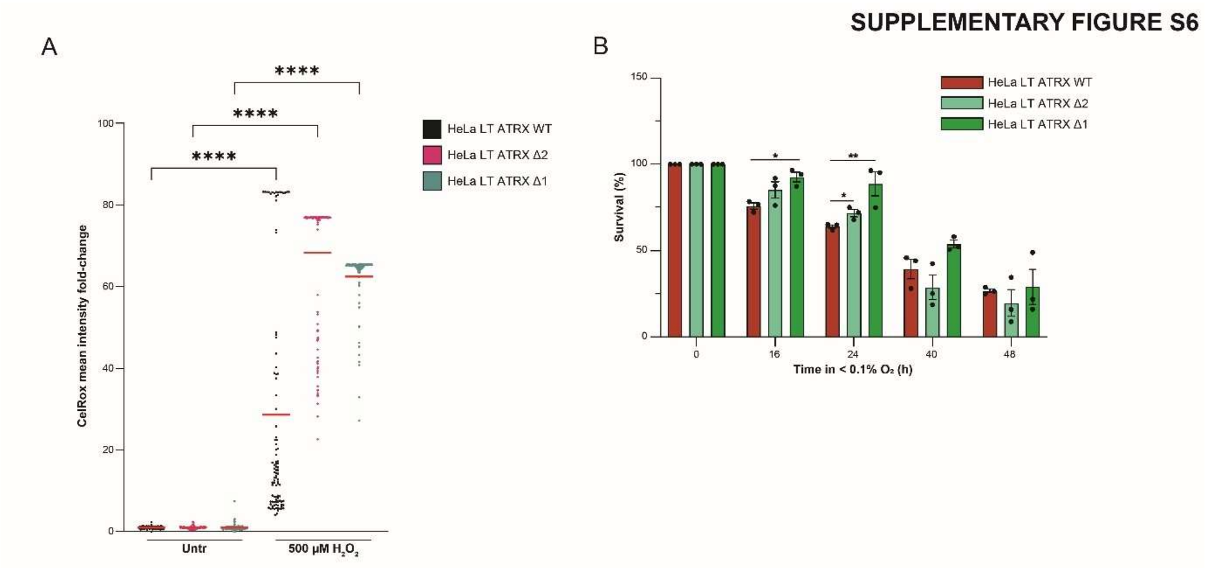

Figure S6: Loss of ATRX confers a small survival advantage in radiobiological hypoxic conditions. (**A**) CellRox assay showing that all cell lines had elevated ROS levels after H_2O_2 treatment. (**B**) Colony survival assay demonstrating a modest survival benefit of ATRX-knockout cells under conditions of radiobiological hypoxia (< 0.1% O_2).

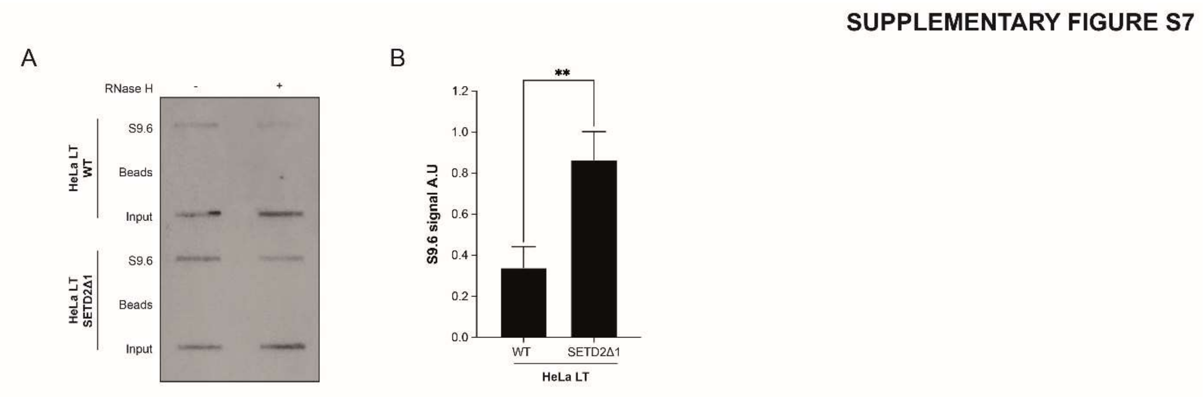

Figure S7: Loss of SETD2 leads to an increase in telomeric DNA:RNA hybrids. (**A**) Representative blot of an S9.6 DNA:RNA immunoprecipitation (DRIP), demonstrating higher levels of DNA:RNA hybrids in telomeric DNA of SETD2-knockout HeLa LT cells as compared to wildtype cells (**B**) Quantification of (A), 3 biological replicates, unpaired t-test.

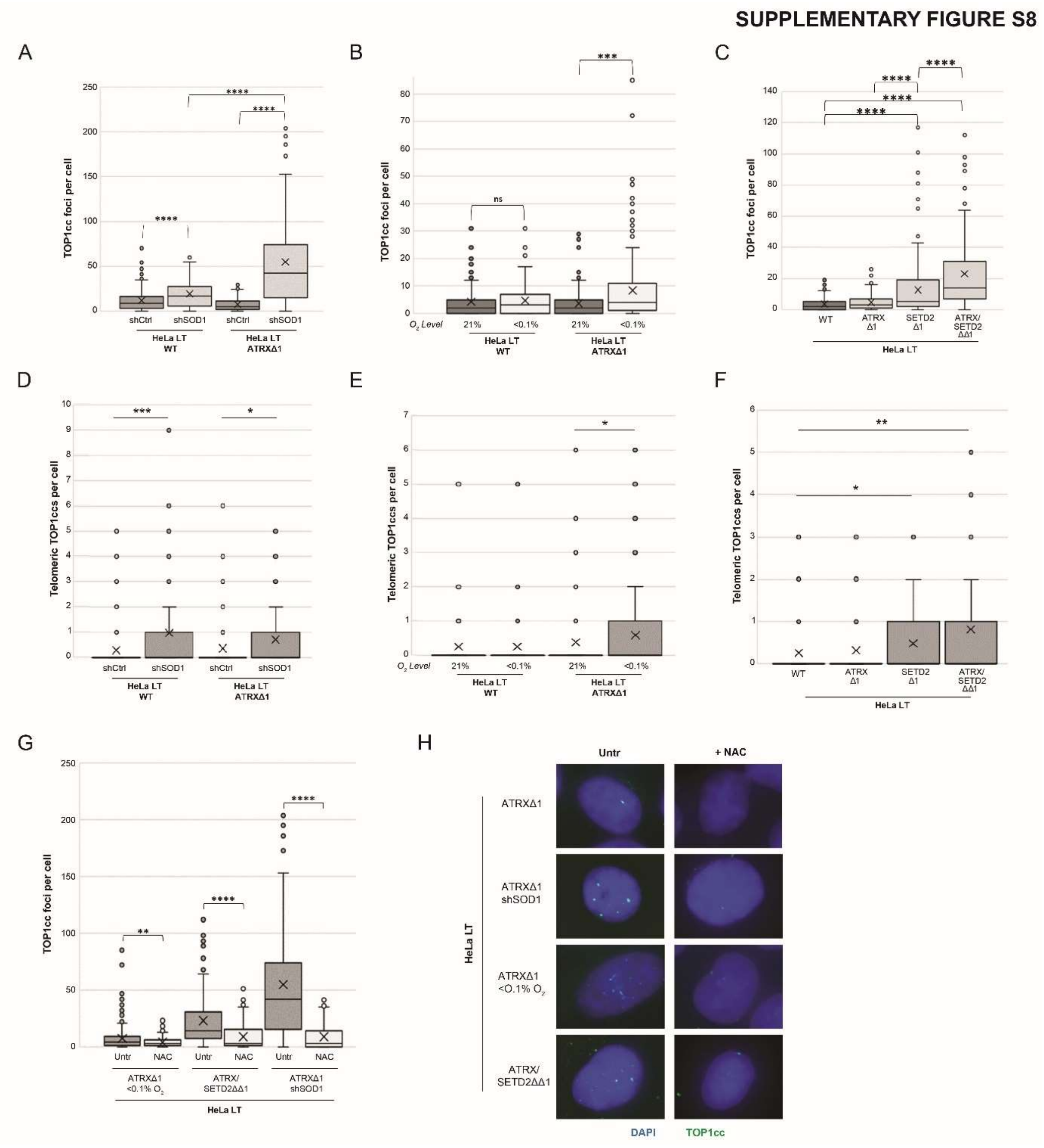

Figure S8: Excessive reactive oxygen species are associated with increased TOP1cc formation. (**A-C**) Quantification of global trapped TOP1 protein (TOP1cc) upon silencing of SOD1 gene expression (**A**), exposure to radiobiological hypoxia for 6 hours (**B**) or knockout of ATRX/SETD2 (**C**), >100 cells analysed for each condition, Kruskall-Wallis test. (**D-F**) Quantification of telomeric trapped TOP1 protein (TOP1cc) upon silencing of SOD1 gene expression (**D**), exposure to radiobiological hypoxia for 6 hours (**E**) or knockout of ATRX/SETD2 (**F**), >100 cells analysed in each of 3 biological replicates for each condition. (**G-H**) The observed increase in trapped proteins was reversed by treatment with NAC; quantification (**G**) and representative images (**H**), >100 cells analysed for each condition, Kruskall-Wallis test.

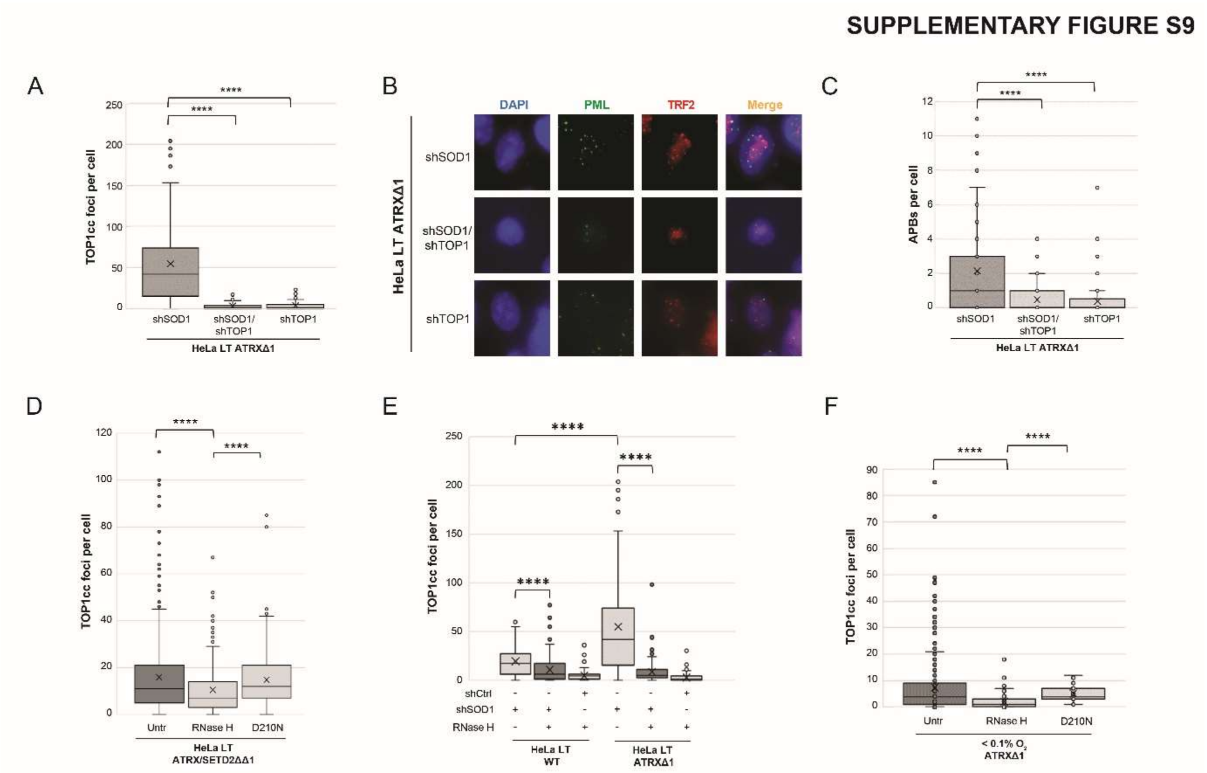

Figure S9: Excessive reactive oxygen species generate R-loops, and this might be linked to DPC formation. (**A**) Control assay demonstrating that accumulation of TOP1cc upon SOD1 knockdown was abolished with concurrent silencing of TOP1. Note that the data for shSOD1 is the same dataset as that presented in Figure S8A. (**B**) Representative immunofluorescence images showing that the observed induction of APBs upon silencing of SOD1 was diminished by co-treatment with shTOP1. (**C**) Quantification of (B), >100 cells analysed for each condition, Kruskall-Wallis test. (**D-F**) Overexpression of RNase H1 was associated with a significant, decrease in levels of TOP1cc under all conditions tested – SETD2 knockout (**D**), shSOD1 (**E**) and radiobiological hypoxia (**F**), >100 cells analysed for each condition, Kruskall-Wallis test.

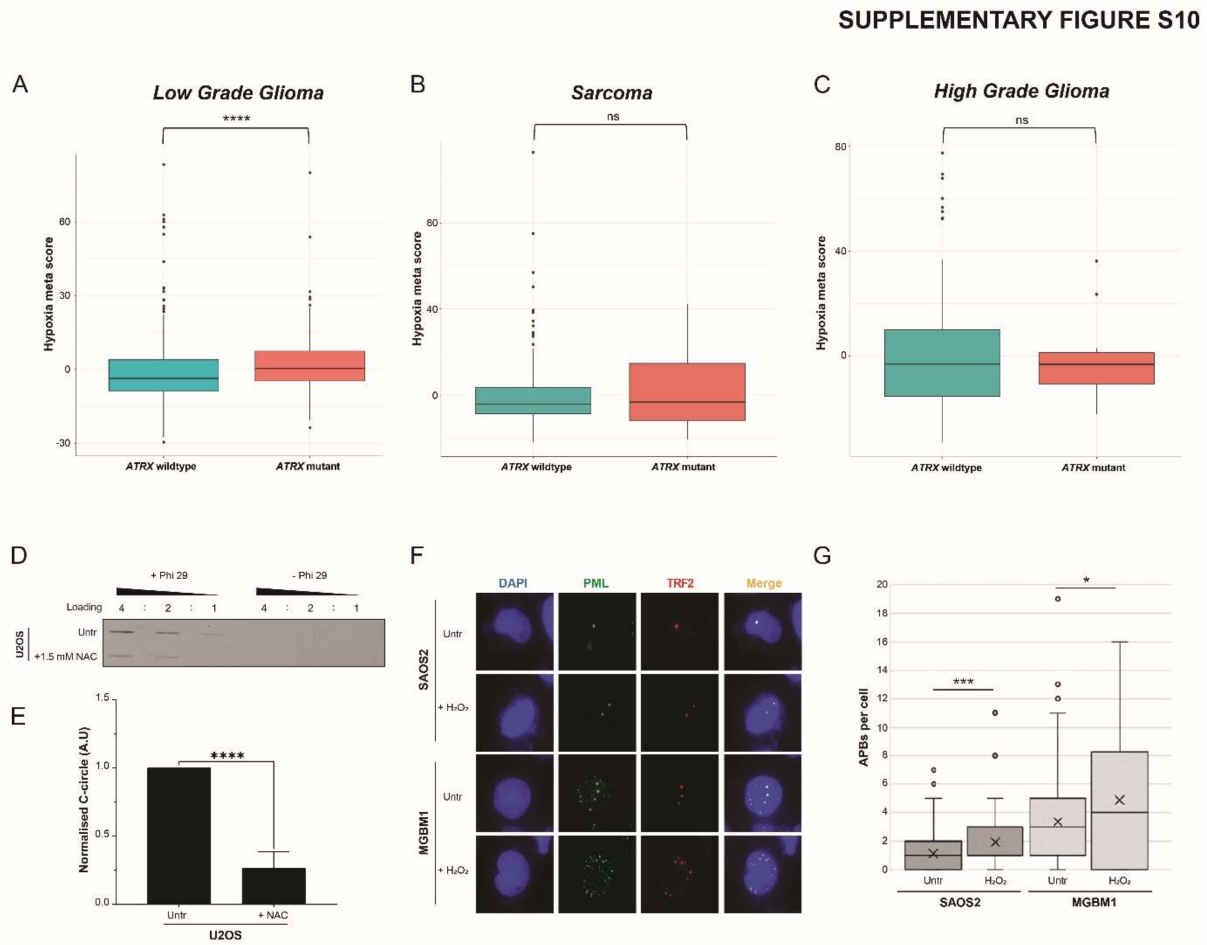

Figure S10: Levels of reactive oxygen species modulate ALT activity in ALT-positive cell lines (**A-C**) HIF metagene expression in RNA-seq analysis was used to calculate a hypoxia metascore for sample from the TCGA-LGG (**A**), TCGA-SARC (**B**) and TCGA-HGG (**C**) datasets. (**D**) Representative C-circle assay blot of U2OS cells treated with the antioxidant NAC. (**E**) Quantification of (D), 2 biological replicates, unpaired t-test. (**F**) Representative immunofluorescence images showing that treatment of ALT-positive SAOS2 and MGBM1 cell lines with 100 µM H_2O_2 leads to a significant increase in the frequency of APBs. (**G**) Quantification of (F), >100 cells analysed for each cell line, Kruskall-Wallis test.

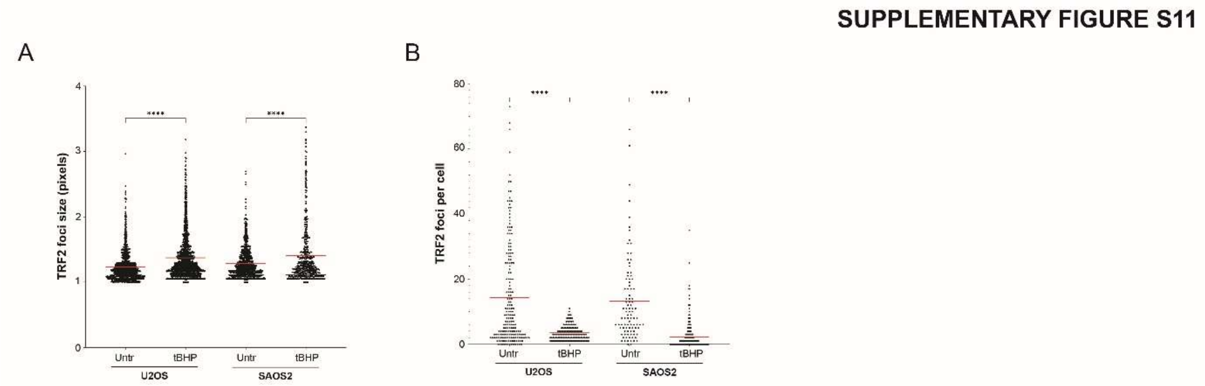

Figure S11: Clustering of ALT telomeres following tBHP treatment. (**A**) Quantification showing an increase in TRF2 foci size in U2OS and SAOS2 cells treated with 10 µM tBHP for 48 hours, > 100 cells analysed for each condition, unpaired t-test. (**B**) Quantification showing a decrease in TRF2 foci per cell in U2OS and SAOS2 cells treated with 10 µM tBHP for 48 hours, > 100 cells analysed for each condition, unpaired t-test.
